# A Review on Covalent Organic Frameworks as Artificial Interface Layers for Li and Zn Metal Anodes in Rechargeable Batteries

**DOI:** 10.1002/advs.202308087

**Published:** 2023-12-08

**Authors:** Yunyu Zhao, Kaiyong Feng, Yingjian Yu

**Affiliations:** ^1^ College of Physics Science and Technology Kunming University Kunming Yunnan 650214 China

**Keywords:** anode dendrite, covalent organic framework, Li metal batteries, synthesis method, Zn ion batteries

## Abstract

Li and Zn metals are considered promising negative electrode materials for the next generation of rechargeable metal batteries because of their non‐toxicity and high theoretical capacity. However, the uneven deposition of metal ions (Li^+^, Zn^2+^) and the uncontrolled growth of dendrites result in poor electrochemical stability, unsatisfactory cycle life, and rapid capacity decay of batteries assembled with Li and Zn electrodes. Owing to the unique internal directional channels and abundant redox active sites of covalent organic frameworks (COFs), they can be used to promote uniform deposition of metal ions during stripping/electroplating through interface modification strategies, thereby inhibiting dendrite growth. COFs provide a new perspective in addressing the challenges faced by the anodes of Li metal batteries and Zn ion batteries. This article discusses the stability and types of COFs, and summarizes some novel COF synthesis methods. Additionally, it reviews the latest progress and optimization methods of using COFs for metal anodes to improve battery performance. Finally, the main challenges faced in these areas are discussed. This review will inspire future research on metal anodes in rechargeable batteries.

## Graphical abstract

This article discusses the stability and types of COFs, and summarizes some novel COF synthesis methods. Additionally, the recent progress of COFs as a protective film for rechargeable Li and Zn anodes is reviewed. Finally, some optimization strategies for COFs used in Li and Zn anodes are suggested, and the main challenges faced in these areas are discussed.

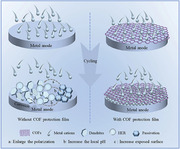

## 1 Introduction

**Figure 1:**
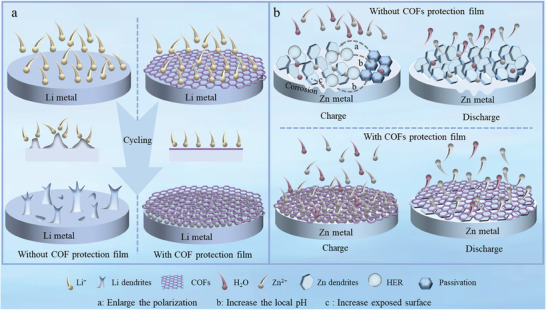
The comparison of bare metal anode (Li, Zn) and COFs@metal anode (Li, Zn) for rechargeable batteries after cycling.

The rapid consumption of fossil fuels and their adverse effect on the environment have resulted in the advancement of renewable energy sources, including geothermal energy, wind energy, and solar energy. These energy sources are important for achieving the vision of global sustainable development.^[^ ^1^, ^2^ ^]^ However, the inherent intermittency and instability of these energy sources have greatly limited their applications.^[^ ^3^, ^4^, ^5^ ^]^ Rechargeable batteries are effective energy storage technologies that can store supernumerary energy generated by renewable energy sources for convenient use.^[^ ^6^, ^7^ ^]^ Therefore, studies on rechargeable batteries mainly focus on energy storage systems. At present, rechargeable batteries have been widely used in electric vehicles, medical devices, electronic products, and military equipment because of the successful commercialization of Li‐ion batteries (LIBs) in the 1990s.^[^ ^8^, ^9^, ^10^, ^11^ ^]^ Since the commercialization of LIBs, they have dominated the market for decades, particularly in electric vehicles and portable electronic products, because of their advantages, including high efficiency, long lifespan, and low maintenance costs.^[^ ^12^, ^13^, ^14^ ^]^ However, traditional LIBs are expensive and have low energy density, thus limiting their applications.^[^ ^15^, ^16^, ^17^, ^18^ ^]^ Li metal is deemed the “ultimate negative electrode” for Li batteries owing to its superiorities, including lightweight (0.53 g cm^−3^), low electrochemical potential (−3.04 V), and excellent theoretical capacity (3860 mAh g^−1^).^[^ ^19^, ^20^, ^21^, ^22^ ^]^ LMBs are mainly composed of cathode, membrane, anode, and electrolyte.^[^ ^23^, ^24^, ^25^ ^]^ LMBs store and convert electrical energy through the round‐trip movement of Li^+^ between negative and positive electrodes. During the discharge process, Li^+^ migrates from the anode to the cathode, releasing electrons that flow back to the positive electrode material through an external circuit, completing the circuit closure. In this process, the internal chemical energy of the battery is converted into electrical energy for use through external circuits. During the charging process, current flows into the battery from an external power source, Li^+^ in the positive electrode material migrates to the negative electrode material through the electrolyte, and electrons flow back to the negative electrode material through the external circuit. Li^+^ recombines with electrons to form Li metal.^[^ ^26^, ^27^, ^28^, ^29^ ^]^ During the recombination process, uneven deposition of Li^+^ on the negative electrode will cause uncontrolled dendrites growth, which may result in internal short circuits or even explosions in the battery (**Figure** **1**a). The detached dendrites can also form “dead Li”, which can reduce the Coulombic efficiency (CE) of LMBs and foreshorten their cycle life.^[^ ^30^, ^31^, ^32^, ^33^ ^]^ In addition to Li metal anodes (LMAs), other metal anodes based on electroplating/stripping electrochemistry, such as Zn, Mg, Na, and K, can also be utilized to construct high energy density secondary metal batteries thanks to their low cost, low electrochemical potential, satisfactory theoretical specific capacity, and excellent electronic conductivity.^[^ ^34^, ^35^, ^36^ ^]^ Among them, Zn ion batteries (ZIBs) have gained considerable attention as a type of secondary battery because of their advantages of lower cost and high safety.^[^ ^37^ ^]^ However, Zn metal anodes have similar issues to LMAs, such as dendritic growth. Zn dendrite growth intensifies the side reactions (i.e., hydrogen evolution reactions (HER) and corrosion) by expanding the exposed Zn anode area. The dendrite growth also reduces ZIB capacity and increases the impedance (Figure 1b).^[^ ^38^, ^39^, ^40^ ^]^ Moreover, the extended interface further stimulates by‐product formation and incubates additional dendrite nucleation sites. These dendrite nucleation sites drive the system into a vicious cycle, leading to battery breakdown and eventual short circuit.^[^ ^41^, ^42^ ^]^ Therefore, researchers have adopted various solutions, including building nucleation layers or scaffolds, optimizing electrolytes or additives, and creating surface protective layers.^[^ ^43^, ^44^, ^45^, ^46^ ^]^ At present, inorganic materials and metal‐organic frameworks (MOFs) have been reported as metal anode protective film. Among them, inorganic materials have high mechanical strength and good ionic conductivity. For example, some studies use inorganic materials (such as Li_3_N, pomegranate Li_6.4_La_3_Zr_1.4_Ta_0.6_‐O_12_, Li_2_S and lithiophilic alloy) as artificial SEI coating on Li surface, which cannot only restrain the stress of dendrite growth, but also provide sufficient Li^+^ migration, and promote the uniform distribution of lithium ions near the anode.^[^ ^47^ ^]^ But they are usually very fragile, and there are some challenges to processing them into inorganic artificial SEI with dozens of microns thickness as a protective film. Using TiN nanosheets with high specific surface area and metal properties as a zinc anode protective layer optimized the electron conduction and Zn^2+^ flux and reduced the polarization of the electrode surface by Jiao et al.^[^ ^48^ ^]^ The AZIB based on the TiN‐coated Zn electrode had a cycle performance with more than 600 cycles and a capacity retention rate of 97.04%. MOFs have been widely used in various metal batteries and other energy storage devices due to their controllable topological structure, large specific surface area, and high thermal stability. For example, Zhang et al. prepared a composite anode by using spin‐coating technology on the Li metal after mixing Zn‐MOF with polyvinyl alcohol (PVA) adhesive, which provided channels and central metal elements to restrict the passage of anions and accelerate the migration of Li^+^.^[^ ^49^ ^]^ The improved electrode showed a more stable CE and longer cycle life expectancy than the bare electrode. Cui et al. constructed porous ZIF‐8 layer on Zn anode by in situ growth method. Zn@ZIF‐8 anode showed low nuclear potential, uniform Zn^2+^ concentration distribution, smooth galvanizing / stripping, and enhanced Zn corrosion resistance at 0.5 mA cm^−2^@ 0.2 mAh cm^−2^.^[^ ^50^ ^]^ Zn@ZIF‐8 symmetric battery extends the battery cycle life to 680 h, which was 2.78 times that of bare zinc foil. However, the disadvantages of high cost and complicated preparation process limit further application of MOFs.

Covalent organic frameworks (COFs) are new organic materials constructed by connecting organic units together through dynamic covalent bonds to form a crystalline and porous structure. COFs are characterized by excellent chemical modification, large surface area, and open porosity.^[^ ^51^, ^52^, ^53^, ^54^, ^55^ ^]^ Compared with traditional inorganic materials, COFs have diverse and controllable structures, making them promising materials for developing high‐performance electrode materials.^[^ ^56^, ^57^ ^]^ Owing to the extension of the skeleton of COFs, they can effectively solve the problem of organic molecule dissolution in electrolytes when used on metal battery anodes and provide larger active sites to improve the specific capacity of the electrode.^[^ ^58^, ^59^ ^]^ In addition, COFs can facilitate the rapid insertion and extraction of metal ions into active centers through ordered directional ion channels and structures, thereby accelerating the diffusion of metal ions.^[^ ^60^, ^61^, ^62^ ^]^ Moreover, owing to the well‐arranged and limited pore size of COFs, metal ions can be rapidly transported through them while limiting and screening other ions and molecules. Their pore sizes help them stabilize the sedimentation of metal ions and restrain the growth of dendrites, thus solving problems, such as HER and corrosion of metal anodes.^[^ ^63^, ^64^ ^]^ Therefore, COFs are considered promising materials for solving the anode problem of metal batteries. In recent years, several studies have focused on fabricating COF protective layers on metal anodes to inhibit dendrite growth, and they have achieved remarkable results. For example, in 2022, Li et al. designed a lithophilic COF containing pre‐organized triazine rings and carbonyl groups as a multipurpose intermediate layer for Li metal batteries (LMBs). When a lithophilic COF layer was introduced to the Li–LFP battery, it retained 86% capacity after 450 cycles at 0.5 C, achieving a capacity of 150 mAh g^−1^.^[^ ^65^ ^]^ In the same year, Wang et al. proposed a collaborative strategy of promoting Zn^2+^ migration dynamics and adjusting surface energy to achieve dendrite‐free Zn deposition, thereby inhibiting self‐corrosion. The deposition of sulfonic covalent organic framework (SCOF) protective layers on Zn foils accelerated the diffusion of Zn^2+^, alleviated the uneven distribution of Zn^2+^, and promoted cation supplementation. Combined with uniform, dense nucleation resulting from improved Zn^2+^ migration behavior, Zn deposits preferentially align in the direction of crystallization (002). Thus, the resistance to dendrites and self‐corrosion during plating/stripping is significantly improved, extending the life of SCOFs@Zn symmetric batteries to more than 4000 and 3000 h at 5 mA cm^−2^@1 mAh cm^−2^ and 5 mA cm^−2^@2 mAh cm^−2^, respectively.^[^ ^66^ ^]^

**Figure 2:**
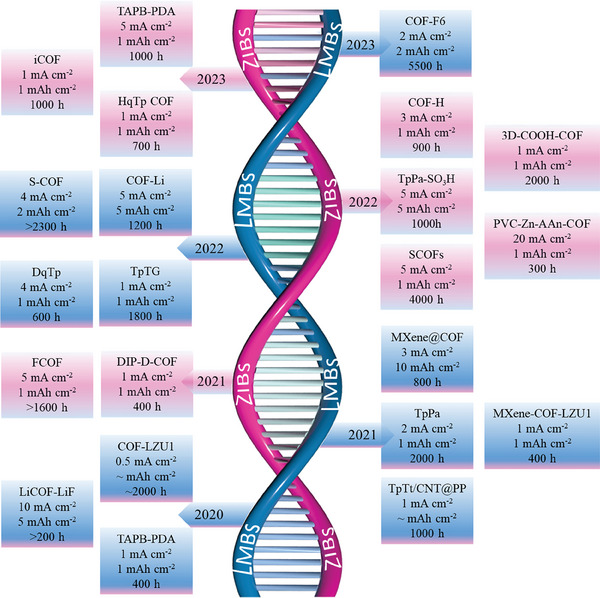
Development of COFs applied for Li and Zn metal anodes in rechargeable batteries.

To date, numerous examples of COFs as an artificial interface layer to protect the Li and Zn anodes of rechargeable metal batteries have been reported in the literature (**Figure** **2**). However, few reports comprehensively discuss this aspect. Therefore, in this essay, we first systematically introduce the stability, types and structures of COFs and summarize their novel synthesis methods. Then, the recent progress of COFs as a protective film for rechargeable Li and Zn anodes is reviewed. Finally, some optimization strategies for COFs used in Li and Zn anodes are suggested, and the prospects for the development of COFs in LMBs and ZIBs are proposed. We hope this essay will provide insight into the advancement of COFs for energy storage.

## 2 Stability, Types, and Synthesis Methods of COFs

Since the first synthesis of boron‐containing COFs in 2005, the development of COF materials has made rapid progress.^[^ ^67^ ^]^ The topological structure of COF materials has expanded from 0D to 3D, and their types and pore sizes range from amorphous to crystalline and micropores to mesopores, respectively. As more COF materials are discovered, their synthesis methods and characterization techniques are constantly being developed to meet different research needs. This development has made COF a new type of functional material, attracting considerable attention in the past few years. Here, we introduce the stability of COF, summarize the classification of COFs from different perspectives, introduce recent novel synthesis methods of COF and the preparation of COF membranes.

### 2.1 Stability

As far as COF itself is concerned, the porous crystal skeleton is connected by covalent bonds through molecular structures. The formation of such covalent bonds is often realized by thermodynamic equilibrium reaction, so it is reversible, which severely limits the stability of COF material. Stability, including chemical, thermal and mechanical stability, is considered one of the prerequisites for the widespread application of COFs. Among them, chemical stability refers to the capability to maintain long‐distance ordered structures in multifarious chemical environments (e.g., acids, bases, redox agents, and solvents), related to the transfer and storage of ions and carriers.^[^ ^68^ ^]^ However, the chemical stability of most COFs is considered the Achilles heel of COFs and fails to keep their structural completeness in harsh chemical environments, thus critically limiting their ion exchange and proton conduction. Fortunately, some strategies have been suggested to construct the high chemical stability of COFs, which greatly expands the application scope of COFs. For example, Li et al. converted imine‐linked COFs into a super‐stable porous aromatic framework via aza‐Diels‐Alder cycloaddition reaction by dynamically fixing reversible imine bonds.^[^ ^69^ ^]^ The formation of quinoline‐linked COFs retained the porosity and crystallinity, and remarkably improved the chemical stability compared with the imino‐COF precursor, making it one of the most stable COFs at present and able to withstand strong acidic, alkaline and redox environment. Thermal stability refers to the ability of COFs to not decompose and work stably under extreme high temperature conditions, which is related to the chemical bond firmness of COFs. By summarizing several cases of using COF as a metal anode protective film, it is found that their thermal stability can basically reach 300 °C. For example, the LiCOF‐LiF prepared by Zhao et al. maintained good thermal stability up to 400 °C without any decomposition.^[^ ^70^ ^]^ Mechanical stability is mainly correlated with the elastic and plastic deformation of the COF membranes. Elastic deformation is a reversible change of the film under the action of external force, which can be recovered after the external force is withdrawn. While plastic deformation often means the formation of defects and the expansion of cracks, with the accumulation of these irreversible deformation, the film will eventually break. Therefore, it would be more stable for the flexible COF membranes with a higher the elastic modulus, while stronger piercing resistance would be beneficial for rigid COF membranes. For example, the COF films prepared by Cheng et al. have high mechanical strength up to 6.8 GPa and exhibit good mechanical stability.^[^ ^71^ ^]^

### 2.2 Types and Structures

**Figure 3:**
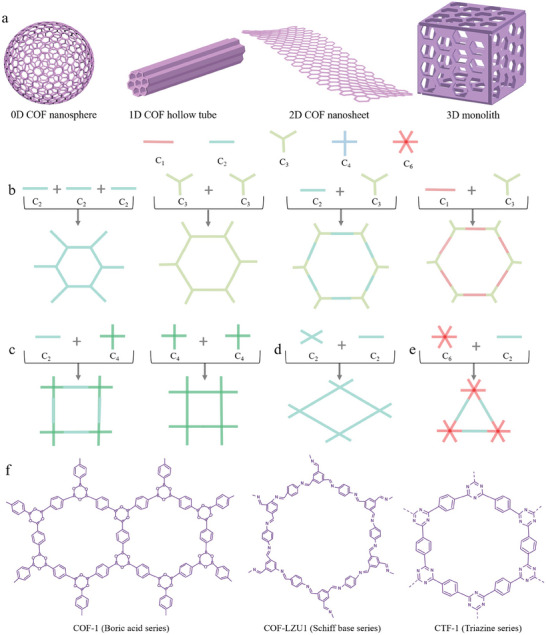
a) COF models for various dimensions; b) hexagonal COF; c) rectangular COF; d) diamond COF; e) triangle COF; f) the different series of COF.

According to the covalent connection framework, COFs can be classified as 0D COFs, 1D COFs, 2D COFs, and 3D COFs (**Figure** **3**a). COFs of different dimensions have different shapes. 0D COFs are spherical or have particle shape; 1D COFs are mainly rod, line, or tube‐like shape; 2D COFs include nanosheet and thin film; 3D COFs are characterized by extended structures, such as bubbles, monolithic blocks, and other layered structures fixed in 3D space. In 2D COFs, covalent bonds exist merely in conjugated 2D sheets, with weak interactions occurring between the layers (such as π‐π packing, hydrogen bonding, and van der Waals forces). In contrast, in 3D COFs, the entire 3D skeleton is linked by covalent bonds.^[^ ^72^ ^]^ As a result, more empty frames are formed in 3D COFs lacking π‐π stacking, making 3D COFs less stable than 2D COFs. In addition, in 3D networks, especially in dia or pts topologies, interpenetration between channels often occurs, eventually leading to the height contraction of channels.^[^ ^73^ ^]^ Although 3D COFs have some challenges, they still have great research and application value because of their unique characteristics. Generally, 2D COFs only have a unified 1D channel, while 3D COFs have a more complex pore structure, including interpenetrating channels and cage structures, which is more favorable in separation, catalysis, and guest incorporation. In addition, compared with 2D structures, 3D COFs have more void frames and easily accessible abundant active sites. They also have high specific surface area and low density, which benefit their practical application. For example, Wu et al. in situ synthesized an ultra‐thin and uniform 3D COOH modified covalent organic framework membrane (3D‐COOH‐COF). The 3D skeleton of the COF membrane and the abundant COOH negative functional groups in the nanochannels accelerated the migration of Zn^2+^ while rejecting the passage of sulfate ions through the protective membrane. The COF membrane not only inhibits the Zn dendrites formation by increasing the transfer number of Zn^2+^ but also reduces the appearance of side reactions by inhibiting the transport of anions.^[^ ^74^ ^]^

As mentioned above, COFs of different dimensions have different shapes. At the same time, COFs of the same dimension also have different shapes. In particular, 2D COF has a variety of sheet structures, including hexagonal, square, diamond, and triangular structures.^[^ ^75^ ^]^ The COFs of these different structures are formed by combining the building units with different symmetries (e.g., C_1_, C_2_, C_3_, C_4_, and C_6_) in a specific way. Among the structures, hexagonal channels are the most common 2D covalent organic frame materials. The two COF materials earlier reported, namely COF‐1 and COF‐5, have hexagonal channels.^[^ ^63^ ^]^ The COF hexagonal structure is obtained by combining the symmetries of C_1_, C_2_, and C_3_ as (C_2_+C_2_+C_2_), (C_3_+C_3_), (C_2_+C_3_), (C_1_+C_3_) (Figure 3b). For example, NTU‐COF‐2 is derived from TAPB (C_3_ symmetry), FPBA (C1 symmetry), and HHTP (C_3_ symmetry) reactions.^[^ ^76^ ^]^ In contrast, COF‐1 and CTF‐1 are synthesized through the self‐polycondensation of p‐phenyldiboric acid (C_2_ symmetry) and 1, 4‐dicyanobenzene (C_2_ symmetry), respectively.^[^ ^67^ ^]^ The COF of the square structure is obtained by reacting (C_4_+C_2_) and (C_4_+C_4_) building blocks with C_2_ and C_4_ symmetries (Figure 3c). For example, NiPc COF is obtained by reacting (OH)_8_PcNi (C_4_ symmetry) with BDBA (C_2_ symmetry), while H_2_TPP‐NIPC‐COF is obtained by reacting H_2_TPP (C_4_ symmetry) with (OH)_8_PcNi (C_4_ symmetry).^[^ ^77^, ^78^ ^]^ The diamond‐structured COF can be constructed in two ways (Figure 3d). The first is the reaction of C_2_ symmetrical rectangular building units with C_2_ symmetrical linear building units. For example, Py‐Azine COF comprises TFPPy (C_2_ symmetric, rectangular) and hydrazine (C_2_ symmetric, rectilinear) reaction system.^[^ ^79^ ^]^ Second, COF can be obtained by reacting two rectangular building blocks with C_2_ symmetry. For example, TTF‐Py‐COF is synthesized by reacting 2,3,6,7‐tetra (4 formylphenyl) tetrathiafulvalene (C_2_ symmetry, rectangle) with 1,3,6,8‐tetra (4‐aminophenyl) pyrene (C_2_ symmetry, rectangle).^[^ ^80^ ^]^ The COF triangular structure is relatively rare and is obtained through the reaction of a hexagonal building block with C_6_ symmetry and a rectilinear building block with C_2_ symmetry, such as the reaction of HEX (C_6_ symmetry) with hydrazine (C_2_ symmetry) (Figure 3e).^[^ ^81^ ^]^ In addition, some COFs can exhibit multiple shapes at the same time. For example, in 2014, Zhao et al. synthesized a dual‐core COF with both triangular and hexagonal pores by reacting C_2_ symmetric rectangular monomer 4, 4 ‘, 4′ ‘, 4′ ‘‐ (ethene‐1,1,2,2‐tetrayl) tetraniline (ETTA) with C_2_ symmetric linear monomer against benzaldehyde.^[^ ^82^ ^]^

In addition, common COFs can be roughly divided according to the different linking groups. Thus, COFs can be categorized into boric acid series, Schiff base series, and triazine series (Figure 3f). The boric acid series includes boric anhydride and borate. The dehydration of boric acid to form boric anhydride (boroxane) and the dehydration condensation of boric acid with ortho‐diphenol to form borate ester were the first successful reactions used to synthesize COFs. Moreover, the first type of COFs developed by Yaghi et al. in 2005 was synthesized through dehydration and self‐condensation of p‐phenyldiboronic acid to form a boronic anhydride as the connecting group.^[^ ^72^ ^]^ The reaction of the boric acid series to synthesize COFs is highly reversible; as a result, the synthesized COFs usually have high crystallinity. However, due to the low electron properties of boron, the boric acid series of COFs are more sensitive to moisture, making them completely decompose under ambient humidity. Therefore, the chemical stability of COFs synthesized through the above reaction is restricted, greatly limiting their practical applications. Thus, the Schiff base COFs were developed. Yaghi et al. were the first to synthesize COFs of the Schiff base series using tetrahedral tetra‐(4‐anilyl) methane and para‐phenyldiformaldehyde with linear structure through dehydration condensation. The synthesized Schiff base series COF was named COF‐300, and it was a 3D COF connected by imine bonds.^[^ ^83^ ^]^ COF‐300 has a 3D diamond‐like skeletal structure, high specific surface area, and better chemical stability than boric acid series COFs but slightly lower crystallinity.^[^ ^84^ ^]^ In comparison, the imine series COFs synthesized through the reaction of anhydride and amino group have better crystallinity. However, few examples of this series of COFs successfully synthesized have been reported due to the low reversibility of the reaction required to form polyimides. In addition, the triazine series of COF materials are synthesized through cyclic trimerization of aromatic nitrile compounds in molten Zn dichloride at 400°C. Due to the high temperature required for the reaction, the finite monomers suitable for this kind of reaction, and the low crystallinity of triazine COF materials, the development and application of the triazine series of COF are greatly restricted. However, triazine COFs have good thermal and chemical stability and high nitrogen content, making them promising in heterogeneous catalysis and other fields.

### 2.3 Synthesis Methods

Generally, the solvothermal method is extensively used to synthesize COFs. However, this method requires a long reaction time and consumes high energy input. In 2009, Cooper et al. used a microwave‐assisted solvothermal approach to synthesize COF‐1 and COF‐5. The rate was 200 times that of the traditional solvothermal method, and the specific surface area of COF‐5 was apparently enhanced to 2109 m^2^ g^−1^.^[^ ^85^ ^]^ The microwave‐assisted solvothermal method greatly increases the reaction rate, increases the yield, and reduces the energy consumption. However, this method is still limited by several issues, such as harsh reaction conditions and high operational difficulty. Therefore, to synthesize a variety of COFs and make them more universal, researchers have explored a variety of assisted solvothermal synthesis methods for COFs. Some conventional synthesis methods, including solvothermal synthesis, mechanochemical synthesis, ionic thermal synthesis, microwave adjuvant thermal synthesis, and interface synthesis, have been discussed in the previous article.^[^ ^86^ ^]^ In this section, we mainly discuss some synthetic methods for synthesizing COFs with excellent properties under certain conditions.

#### 2.3.1 Multicomponent Reactions (MCRs)

**Figure 4:**
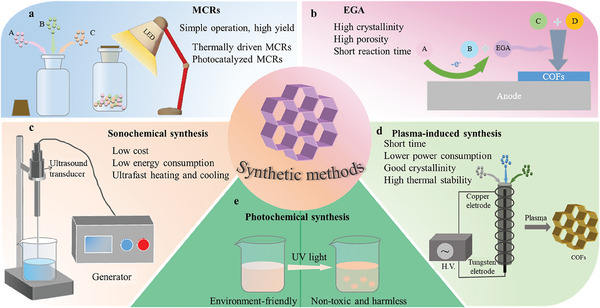
Different synthesis methods of COFs.

Multicomponent reactions (MCRs) are one‐pot reactions that directly admix three or more reactants to synthesize the desired products (**Figure** **4**a).^[^ ^87^ ^]^ MCRs offer a significant advantage in chemical synthesis by reducing the number of reaction steps through a one‐pot synthesis route. “One‐pot” reactions carried out in a single reaction vessel are highly efficient and reduce waste. In addition, the in situ prepared intermediates can be rapidly converted into products without any significant accumulation of reactants, thereby eliminating the need to treat harmful and active intermediates, which is another characteristic of green and sustainable systems. In particular, these advantages make MCRs a promising method for constructing stable crystalline COFs by uniting various reversible/irreversible covalent components. Furthermore, MCRs include thermally driven MCRs and photocatalyzed MCRs. In contrast to thermally driven MCRs, photocatalytic MCR synthesis of COFs is less studied. Photocatalyzed MCRs have numerous advantages, including simplicity, high synthetic efficiency, and diversity, compared with traditional chemical reactions. Recently, Wang et al. applied the polymerization strategy based on photocatalytic MCRs to synthesize COF for the first time.^[^ ^87^ ^]^ Under visible light irradiation, they condensed potassium cyclohexyl trifluoroborate with different hydrazides, amines, and aldehydes. Under environmental conditions, they successfully synthesized a variety of robust COFs with high crystallinity and everlasting porosity through photooxidation‐catalyzed multicomponent Petasis reactions. Among them, the CyN_3_‐COF exhibited high photocatalytic activity and recyclability in the visible light oxidation hydroxylation of arylboric acid. Therefore, photocatalytic synthesis based on MCRs not only enriches the synthesis methods of COFs but also provides a viable green approach to obtaining COFs with different functions.^[^ ^88^ ^]^

#### 2.3.2 Electrogenerated Acid (EGA)

Currently, imido COFs are mostly synthesized at ordinary temperature using Lewis acid catalysts or under solvothermal conditions with glacial acetic acid and water.^[^ ^89^ ^]^ The COF powders generated through these synthesis methods are indissoluble in most organic solvents, making COFs difficult to form and fix when used on the substrate, causing great inconvenience. To solve this problem, Shirokura et al. proposed a synthesis method called electrogenic acid (EGA) (Figure 4b).^[^ ^90^ ^]^ This method uses EGA that is produced on the electrode surface through electrochemical oxidation of appropriate precursors as an efficacious Brønsted acid activator, which enables the corresponding aldehyde and amine monomers to form imine bonds while deposing corresponding COF films on the electrode surface. For example, they synthesized imido COF film bottom‐up on the electrode using EGA as an activator for the condensation reaction of aldehyde monomers and amine. Due to the low oxidation potential of the EGA source in the reaction system, 1,2‐diphenylhydrazine (DPH) was selected as the EGA source. The COF structures prepared through this method have excellent crystallinity and high porosity, and the membrane thickness can be dominated by the electrolysis time. EGA has proven to be an efficient approach for depositing COFs on the electrode and an innovative synthesis method based on sustainable electrosynthesis. In addition, the use of EGA is characterized by minor environmental effect, short reaction period under mild conditions, and a simple purification process. This new synthesis method is a promising technology for the functional modification of electrodes and sensor parts based on COFs. Moreover, the method can also be applied to synthesize triazine‐based carbon nanotubes and 3D carbon nanotube structures.

#### 2.3.3 Sonochemical Synthesis

Ultrasonic chemical synthesis is an ultrasonic‐assisted chemical reaction using frequencies ranging from 20 kHz to ≈1 MHz (Figure 4c).^[^ ^91^ ^]^ It is a new synthesis method with improved reaction rate and product conversion. Because the energy of the ultrasonic wave is relatively low, this method cannot change the rotational, vibrational, or electronic state of the molecule. Therefore, its chemical effects are caused by acoustic cavitation, which can accelerate the crystallization rate. Ultrasonic‐assisted chemical reaction includes the formation, growth, and implosion of bubbles as ultrasonic waves pass through a liquid medium. The violent implosion of these bubbles produces ephemeral extreme conditions, such as extremely high local pressures and temperatures (≈1000 bar and ≈5000 K), rapid heating and cooling rates (10^10^ K S^−1^).^[^ ^92^, ^93^, ^94^ ^]^ This method is characterized by low energy consumption and cost, and it eliminates the induction period, thus significantly reducing synthesis time. In 2022, Cooper et al. proposed a general acoustochemical method for synthesizing imine COFs in acidic aqueous media.^[^ ^95^ ^]^ A 5050 W sonar operating at 20 kHz and 50% power was used to successfully synthesize one 3D COF and eight 2D COFs in 1 h. The porosity and crystallinity of these COFs are superior to those prepared using the solvothermal method. However, the full potential of acoustochemical synthesis has yet to be unlocked. Although laboratory‐scale acoustochemical reactors are commercially available, relatively few equipment options are used for industrial‐scale manipulations due to a lack of standardized protocols.

#### 2.3.4 Plasma‐Induced Synthesis

In addition to solid, liquid, and gas states, there is a fourth state called plasma. Plasma is a form of matter with charged ions and free electrons as its main ingredients, and it has a relatively high electrical conductivity. It is also known as the “supergas”. Plasma discharges, which contain free electrons, photons, free radicals, metastable species, and excited neutral substances, can be used to initiate chemical reactions.^[^ ^96^ ^]^ Non‐thermal plasmas produce reactive substances at approximately room temperature and atmospheric pressure and have been extensively used to synthesize nanomaterials and polymers (Figure 4d). Liquid dielectric barrier discharge (DBD) plasma is a typical plasma that operates in an atmospheric non‐equilibrium state. DBD plasma has the superiorities of a simple structure and energy conservation, and it can be operated at normal temperature and pressure. It can promote many chemical reactions that are not prone to occur under other conditions.^[^ ^97^ ^]^ For example, in 2021, Hou et al pioneered the synthesis of plasma‐induced COFs using liquid DBD plasmas.^[^ ^98^ ^]^ Under environmental conditions, ten various 2D and 3D COFs were prepared within 1 h. Those COFs have different connections, such as boronate ester‐linkage, azine, β‐ketoenamine, imine, and boroxine, but they can also be tuned directly to the interlayer stacking of COF crystals within few minutes. Moreover, due to the high electron temperature generated by the DBD and the acidic and abundant active radicals of the plasma, COFs can rapidly nucleate and grow under the plasma conditions, resulting in good crystallinity, morphology, and surface area.^[^ ^99^ ^]^ Compared with traditional methods, this method takes less time, consumes less power, and requires no additional heating, inert gas protection, or pressure.

#### 2.3.5 Photochemical Synthesis

Compared with the usual chemical reagents, light is undoubtedly an environmentally friendly, non‐toxic, and harmless renewable energy. Thus, it is generally considered by scientists to be an indispensable reagent in green synthetic chemistry. Photochemical synthesis of COFs uses natural light or ultraviolet light to irradiate reagents or monomers required to synthesize COFs (Figure 4e). The light may act as a catalyst in some reactions and as a reagent in others. Light absorption can promote the participation of electronic excited states during the reaction process of a compound. Excited molecules are better electron acceptors and electron donors than ground‐state molecules. Therefore, their redox properties are different. Photoactivation of organic molecules can be achieved through energy or electron transfer processes.^[^ ^100^ ^]^ For example, Choi et al. photochemically synthesized COF‐5 with a monomer solution exposed to UV‐visible light in a quartz vial, and 75% of the COF‐5 production was achieved within one hour.^[^ ^101^ ^]^ The obtained COF had a distinctive sea urchin shape and Brunauer‐Emmett‐Teller specific surface area up to 2026 m^2^ g^−1^. Density functional theory (DFT) calculations indicated that the out‐of‐plane growth rate in the photochemical process significantly increased, which was attributed to the improved interlayer orbital coupling. After two years, they applied this photochemical synthesis strategy to rapidly synthesize pyrazine‐COF (hcc‐COF) through the condensation reaction of 1,2,4,5‐benzenetetramine (BTA) and hexaketocyclohexane (HCH) under simulated sunlight (wavelength 200–2500 nm). The hcc‐COF was synthesized within 3 h.^[^ ^102^ ^]^ In the absence of illumination reaction, only amorphous polymers were produced, reflecting the important role of light irradiation. Due to the regularity of hcc‐COF fully fused skeleton and structure, the volume conductivity of the hcc‐COF pellets was 2.22 × 10^−3^ S m^−1^, which was the highest among reported COFs. Advances in photochemical synthesis have opened up new paths to synthesize organic chemicals.^[^ ^103^ ^]^ Unfortunately, it is not the best choice in industry on account of the high price of photons, and it is not possible in many laboratories.

### 2.4 Preparation Method of COF Membranes

In recent years, a great deal COF cases have been reported as a metal anode protective film, such as TpPa, FCOF, COF‐H, etc. Their preparation methods are also varied. As a heterogeneous modified layer, a slight difference in the preparation has a significant effect on the electrochemical properties of the metal anode. Therefore, as a metal anode protection layer, the preparation process for COF films is crucial. At present, the preparation methods of protective COF films for Li and Zn anodes can be divided into ex situ preparation methods and in situ preparation methods (**Table** **1**). Ex situ preparation methods included doctor blading method, spin‐coating method and liquid‐liquid interface method.^[^ ^104^ ^]^ Doctor blading is the most commonly used method for preparing ex situ coatings. This method is to combine the COFs with the adhesive in an organic solvent. The slurry is subsequently cast on the substrate, and the thickness is dominated by the blade. A thin COF film would be formed on the surface of the substrate after the solvent evaporates. Among them, N‐Methylpyrrolidone (NMP) is often used as a solvent, and polyvinylidene difluoride (PVDF) is often used as an adhesive. For example, Wang et al. added TAPB‐PDA COF powder, Ketjen Black and PVDF to NMP at a mass ratio of 6:3:1 to form a uniform slurry and scraped it onto the Zn sheets.^[^ ^105^ ^]^ After drying for 12 h in vacuum, the uniform COF protective film was obtained on Zn. This method is extensively applicable and economically feasible owing to its simplicity and maturity in other fields such as electrode preparation in LIBs. Spin‐coating method also uses similar solvents and adhesives as doctor blading method. In this method, the slurry is deposited on the surface of the substrate placed in the center of the turntable, and then the turntable is given a rotation speed. The slurry will flow into all directions to the edge of the substrate at the same time. The COF films preparation is finished when the whole substrate is coated with the slurry. The thickness of the membrane is inversely proportional to the cubic root of the solvent viscosity and the square root of the rotational velocity. Thus, the key factor in its preparation is the rotational speed and viscosity of the matched solvent. In 2020, COF‐LZU1 was successfully synthesized on the Li surface using this method by Xu et al.^[^ ^106^ ^]^ The liquid‐liquid interface method is the process of dissolving one monomer in the organic phase, another monomer and acid catalyst in the aqueous phase, or acid catalyst in the aqueous phase and two monomers in the organic phase, allowing the monomer diffusion reaction at the organic‐aqueous interface to obtain the COF membrane. Zhao et al. dropped 2,4,6‐Triformylphloroglucinol (Tp)‐hexane saturated solution (3 mL) into an aqueous solution (20 mL) containing 2,5‐diamino benzene sulfonic acid (Pa‐SO_3_H, 1 mm) and toluene sulfonic acid (PTSA, 1.5 mm), and the static reaction lasted at room temperature for 3 days.^[^ ^107^ ^]^ A homogeneous TpPa‐SO_3_H film was formed at the two‐phase interface, and then the resultant film was transferred onto a zinc foil or other substrates used for battery assembly. The liquid‐liquid interface method is suitable for preparing large area COF membrane, and the membrane thickness can be controlled by the monomer concentration, but the simultaneous reaction of the monomer at multiple sites at the liquid‐liquid interface can affect the uniformity and crystallinity of the COF membrane. In situ preparation of COF films methods include electrochemical deposition and solid‐liquid interface method. Electrochemical deposition is a method in which some special materials are dropped on the Li/Zn to undergo a chemical reaction and aggregate into a film. For example, Chen et al. dissolved certain amounts of p‐phenylenediamine and 2,4,6‐triformylphloroglucinol in 1,2‐dimethoxyethane/1,3‐dioxolane and tetraethylene glycol dimethyl ether, respectively, as solutions 1 and 2, following by transferring 20 µL of solution 1 to the treated Li foil. After volatilizing, they added the same amount of solution 2 to the Li foil and reacted for 12 h. After washing and drying, the COF membrane was successfully synthesized on the Li metal surface.^[^ ^108^ ^]^ Solid‐liquid interface synthesis is the process of soaking the Li/Zn in solution and COF can grow into membranes on the substrate. Aupama et al. dissolved 2,5‐diamino hydroquinone dihydrochloride (Hq) (9.4 mg, 0.044 mmol) and 2,4,6‐triformyl phloroglucinol (Tp) (3 mg, 0.014 mmol) in 40 ml of a solvent including xylene: dimethylformamide : 1, 4‐dioxane (1:2:1, v/v) to synthesis solutions for HqTpCOF. Then, the solution was sonicated for 15 min to form a homogeneous solution.^[^ ^109^ ^]^ Subsequently, the polished zinc foil was placed in the solution and stored for 24 h. The HqTpCOF‐layer without a binder can grow on the Zn surface with thickness of ≈300–400 nm. It can be suggested that it is more suitable to use doctor blading method and spin‐coating method to prepare protective film of COF that needs to be synthesized at high temperature, while liquid‐liquid interface method is more suitable for the synthesis of COF membrane that can be synthesized at room temperature.

**Table 1:** Summary of COF films preparation methods for Li and Zn metal anodes.

Methods		COFs	
		LMBs	ZIBs
Ex situ preparation	Doctor blading method	DqTp, G@COF‐1, TpTt/CNT@PP	SCOFs, COF‐H, PVC‐Zn‐AAn‐COF, TAPB‐PDA, COF 42
	Spin‐coating method	COF‐LZU1	
	Liquid‐liquid interface	LiCOF‐LiF	iCOF, TpPa‐SO_3_H
In situ preparation	Electrochemical deposition	ACOF, COF‐F6, TpTG, S‐COF, COF‐Li, TpPa, TAPB‐PDA	
	Solid‐liquid interface		3D‐COOH‐COF, DIP D COF, HqTpCOF

## 3 Application of COFs on Metal Anodes

### 3.1 LMBs

By analyzing the electrochemical reaction process, the researchers have found that the electrode material plays a pivotal in improving the electrochemical properties of batteries. However, among the numerous metal materials, only a few metals, such as Li, Zn, Na, and K, are suitable for anode materials for batteries. Among them, Li metal is deemed to be the “holy grail” anode material in metal batteries owing to its low electrochemical potential (3.04 V vs SHE) and excellent theoretical specific capacity (3860 mAh g^−1^).^[^ ^110^, ^111^ ^]^ However, the practical applications of LMBs are limited by the unlimited volume changes, high reactivity, and rampant dendrite growth of LMAs during cycling.^[^ ^112^ ^]^ Specifically, the formation of the Li metal anode dendrite consists of three stages.^[^ ^113^, ^114^ ^]^ First, after the battery assembly, the highly active Li will instantly react with the organic solvent in the electrolyte to form the SEI. This dense SEI transiently prevents the further reaction of the Li metal with the electrolyte. However, the naturally formed SEI is insulating, which not only increases the impedance of the battery, but also increases the transmission resistance of Li^+^ and then affects the uniform deposition of Li^+^. Subsequently, the anode shows a severe expansion/contraction during deposition/stripping because lithium metal is a skeleton‐unbound material. Severe expansion/contraction can cause the SEI to experience drastic fluctuations and fragmentation. Finally, the cracks formed by the original bulge and the SEI layer accelerate the growth of local Li^+^ flux, which leads to uneven Li deposition and eventually the formation of large amounts of dendrite. When the dendrite grows to a certain extent, it will fall off and form dead lithium. The detached Li disconnects the contact with the electrode, and electrons can no longer be delivered to the Li electrode to become Li^+^, so “dead lithium” will reduce the relative content of active Li, thus reducing the energy density of the battery.^[^ ^115^ ^]^ Therefore, the underlying cause of the Li dendrite formation is the high reactivity of the LMA. In addition, because of the high reactivity of Li metal, it is prone to react with the organic electrolyte to form inorganic products such as Li_2_CO_3_, LiOH, Li_2_O, Li_3_N, LiF and organic products such as RCOO_2_Li, ROLi, ROCO_2_Li (R is an alkyl functional group).^[^ ^116^ ^]^ These reactions will lead to lower utilization of lithium metal and electrolyte, and will be accompanied by a large amount of gas, resulting in a change in battery volume, easy to cause safety risks of LMBs. In conclusion, the unstable SEI, the growth of Li dendrites, the volume expansion effect of the electrode and the formation of dead Li greatly deteriorate the electrochemical performance of LMBs, which eventually leads to the low coulomb efficiency, short service life and safety risks of the battery. These issues have been studied for over 50 years since Li metal was first used as a negative electrode for Li batteries. Fortunately, a large number of strategies have been reported to tackle the problems faced by metal anodes. As the metal anode closely contacts the electrolyte in the cell, most of these problems are due to the low compatibility of the metal anode or the electrolyte itself, or between the metal anode and the electrolyte. Therefore, strategies for solving these problems also focus on electrolyte optimization and modification of metal anode. Next, the Li metal anode is taken as an example to illustrate the corresponding protection mechanism of each strategy. Electrolyte optimization mainly improves the stability of SEI through solvent design, changing the Li salt and adding additives, while improving the ionic conductivity and promoting the uniform deposition of Li^+^.^[^ ^117^, ^118^ ^]^ Solvent is the main source of SEI formation on the surface of Li anode, and organic carbonate is a widely used electrolyte solvent. The initial SEI component is mainly composed of the reduction product (ROCOOLi) of alkyl carbonate. Under the action of trace water, it will be further converted into Li_2_CO_3_ solvent, thus affecting the stability of SEI.^[^ ^119^ ^]^ Jiang et al. obtained a new type of fluorobenzene diluted highly concentrated electrolysis (FB‐DHCE) by using a low density and low viscosity fluorophenzene (FB) as a bifunctional co‐solvent to dilute the high concentration of electrolyte (HCE).^[^ ^120^ ^]^ FB‐DHCE not only dilutes the high concentration of LiFSI‐based ether electrolyte, but also enhances the interaction between dimethoxymethane (DME) and FSI^−^ around the Li^+^, effectively improving the interface stability. The unique solvation and interfacial chemistry of FB‐DHCE allowed Li to achieve dendrite‐free deposition leading to high CE (up to 99.3%) and extended cycle life (above 500 cycles at 1 mA cm^−2^). Using separate lithium salts usually has various problems, and mixing several Li salts together with the synergistic effect between different Li salts can protect the negative electrode of LMBs. Fu et al. prepared a novel Succinonitrile (SN) ‐based electrolyte material (SN‐DLi‐FEC) using fluoroethylene carbonate (FEC) additives and Li disalt (Li bis(trifluoromethane sulfonimide) (LiTFSI) and Li difluoro(oxalato)borate (LiODFB)).^[^ ^121^ ^]^ Compared with other SN‐based electrolytes such as SN‐SLi‐FEC (single salt combine with FEC), SN‐DLi‐FEC can generate a more durable and stable organic‐inorganic composite SEI rich in LiF and B─F bond on the surface of LMA. Moreover, SN‐DLi‐FEC has good thermal stability, high ionic conductivity and high oxidation resistance. The LiCoO_2_ (LCO) / Li cell assembled by SN‐DLi‐FEC had a specific discharge capacity of over 140 mAh g^−1^ at 10 °C and room temperature, and had a superior thermal safety at a high temperature of 120 °C. In addition to solvent design and changes in Li salts, adding a small amount of functional additives is also an effective way to optimize electrolytes and control the morphology of Li deposition. For example, Chen et al. used polyhexylactone (PCL‐ONO_2_) prepared by acylated diol (PCL‐diol) as a new electrolyte additive to realize the introduction of high concentration NO_3_^−^ into carbonate electrolyte.^[^ ^122^ ^]^ PCL‐ONO_2_ will preferentially react with Li metal to form a bilayer SEI with an internal nitrogen‐rich layer and an external flexible‐rich organic layer. The internal rigid nitrogen‐rich layer can inhibit Li dendrite growth, inhibit solvent penetration, and promote the rapid transfer of Li^+^. Moreover, the outer flexible organic layer can effectively adapt to volume fluctuations and inhibit the exfoliation of the internal inorganic layer during long‐term circulation. In the assembled symmetric batteries with PCL‐ONO_2_ additives, the lithium metal anode can provide an extremely stable cycling performance for 5000 h in the carbonate electrolyte at 1.0 mA cm^−2^ @ 1.0 mAh cm^−2^. The interface modification of the Li metal anode is mainly to limit the reaction by making an artificial SEI on the metal anode to separate the lithium anode and the electrolyte. From the chemical level, different artificial SEI have different functional characteristics to solve the problems of Li anode from different aspects. However, any ideal artificial SEI should have chemical/electrochemical stability and electronic insulation (large bandgap), not react with lithium metal, not dissolve in the electrolyte, and can work stably at a broad range of operating temperatures and voltage. In addition, artificial SEI can be subdivided into organic coating, inorganic coating and organic/inorganic coating. Usually, inorganic artificial SEI have satisfactory electrochemical stability, a higher mechanical modulus, and a more efficacious Li^+^ diffusion pathway. For example, Cui's group used freon R134a as a reagent to coat the LiF on the lithium surface.^[^ ^50^ ^]^ The LiF coating can be used as a chemically stable and high mechanical strength intermediate phase to minimize the corrosion reaction with the carbonate electrolyte and inhibit the dendrite formation. Gaseous freon not only has well‐controlled reactivity and nontoxicity, but also has better permeability compared with solid/liquid reagents. Thus, a dendrite‐free and stable cycle is realized in 300 cycles with a current density of up to 5 mA cm^−2^. However, due to its brittleness, it is prone to fracture under large changes in lithium metal volume, resulting in the failure of Li metal batteries, especially at large current density. For organic artificial SEI, although its mechanical modulus is inferior to inorganic materials and the ionic conductivity is moderate, its high flexibility and superior workability can achieve close contact with the electrode, which can effectually inhibit the growth of dendrites, thus significantly ameliorating the electrochemical performance. Huang et al. designed a rigid and soft hybrid protective layer containing the inorganic ionic conductive component LiF and the organic component PVDF‐HFP, so that the synthetic artificial SEI film has the advantages of high ionic conductivity, high mechanical modulus, and good compatibility with the lithium metal surface.^[^ ^123^ ^]^ In Li–Li symmetric batteries, this composite artificial SEI helped to improve the battery efficiency and extend the cycle life, and showed superior cycle property with capacity retention rate of 80% after 250 cycles in the full cell with LFP as the paired cathode.

COF films also belong to the class of organic artificial SEI. Numerous studies have shown that COFs, as a member of the rapidly developing family of frame materials, show remarkable electrochemical properties using COF artificial SEI films on LMAs.^[^ ^124^, ^125^, ^126^ ^]^ COFs prevent direct touch between the electrolyte and the Li anode, thereby inhibiting the reaction with the electrolyte to a certain extent on account of the high activity of the Li metal. In addition, the ordered channels and abundant porosity of COFs can regulate the flux of Li^+^, making the plating/stripping process more uniform. The regulation of the flux of Li^+^ cannot only inhibit the growth of Li dendrites but also control the large volume changes caused by the irregular plating/stripping of ions.^[^ ^127^, ^128^, ^129^ ^]^ The above advantages of COFs make them a potential material to solve the severe problems related with LMBs. Therefore, the application strategies and examples of COF and its composites in LMBs are reviewed in this section.

#### 3.1.1 COF‐Based Films with High Mechanical Strength

**Figure 5:**
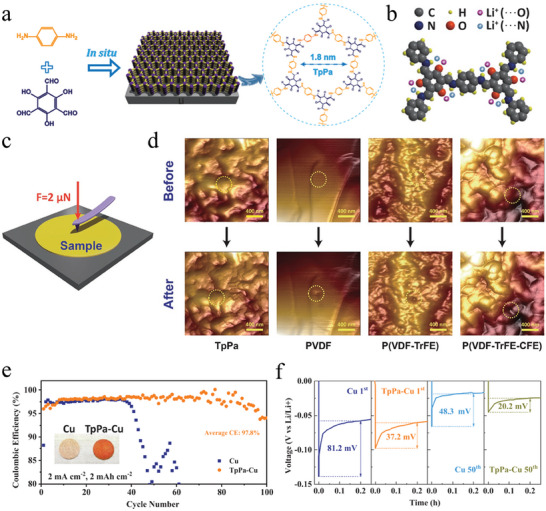
a) Reaction scheme of TpPa from Tp and Pa. b) Lithiophilic sites (N and O) in TpPa. c) Diagram of mechanical test by AFM. d) AFM images before and after puncture by the probe tip with loading force of 2 µN for TpPa, PVDF, P(VDF‐TrFE), and P(VDF‐TrFE‐CFE) films. e) CE of Li||Cu, Li||TpPa‐Cu cells and f) corresponding Li nucleation overpotentials at the first or 50th cycles in (e). Reproduced with permission.^[^ ^108^ ^]^ Copyright 2021, Wiley‐VCH GmbH.

The formation of dendrites inside the battery can shorten the battery life and even cause severe safety problems. These problems can be addressed by regulating the electrolyte composition, optimizing the diaphragm, and designing SEI. Among them, optimizing SEI by improving electrolyte additives can efficiently balance the relation between Li‐ion conductivity and Li dendrite inhibition.^[^ ^130^ ^]^ However, the component of SEI has some influence on the conductivity of the material. Inorganic materials have good ionic conductivity and high mechanical strength. However, their brittleness makes them difficult to be processed into SEI tens of microns thick.^[^ ^131^ ^]^ Organic polymers cannot easily achieve both good ionic conductivity and high mechanical strength at the same time because polymer films with high mechanical strength require high crystallinity, while polymer films with high ionic conductivity require low crystallinity.^[^ ^132^ ^]^ Therefore, designing an LMA protective film with good ionic conductivity and high mechanical strength is necessary to inhibit dendrite growth. Chen et al. prepared TpPa‐COF membranes by in situ reacting 2,4,6‐triformyl‐phloroglucinol (Tp) with p‐phenylenediamine (Pa) at room temperature (**Figure** **5**a).^[^ ^108^ ^]^ TpPa molecules were self‐assembled into a 2D polygonal framework with good solvability, high porosity, electrochemical stability and layered π‐packing structure. When TpPa is used as artificial SEI for LMAs, the evenly arranged and ordered lithiophilic sites in TpPa (Figure 5b) can effectively regulate the Li‐ion flux while also forming a high‐speed channel for Li‐ion flow. In addition, the delocalized electron band equalizes the energies of the groups, thereby effectively raising the Fermi level of the TpPa layer. As a result, a reversible and uniform Li plating/stripping process can be realized to produce high‐quality LMAs. Moreover, the TpPa protective film had a high Young's modulus of 7.25 GPa, effectively inhibiting dendrite formation and exposure and ensuring battery safety. To comprehensively analyze the outstanding performance of the TpPa layer, they studied its mechanical properties using an atom force microscopy (AFM) equipped with digital pulse force mode (Figure 5c). To simulate the transpiercing process of Li dendrites, polyvinylidene fluoride (PVDF), polyvinylidene fluoro‐trifluoroethylene (P(VDF‐TrFE)), polyvinylidene fluoro‐trifluoroethylene (P(VDF‐TrFE‐CFE)) were selected as standard materials for comparison. A force of 2 µN was applied to the rigid AFM probe on each standard material and COF film. As exhibited in Figure 5d, the TpPa membrane remained intact after the puncture, indicating excellent transpiercing resistance. In contrast, in other samples, sinkholes were visible at the point where the tip of the probe was pierced. No significant increase in voltage lag of TpPa‐Li symmetric battery was observed during electroplating/stripping after 2000 cycles. However, the overpotential of the pure lithium symmetric battery continuously increased from the initial 20 mV to ≈200 mV after 600 cycles. This suggests that the TpPa protective film greatly improved the long‐term stability of symmetric batteries. At 2 mA cm^−2^@2 mAh cm^−2^, batteries containing TpPa layers exhibited an average CE of 97.8% after 100 cycles, while the CE on a copper foam substrate quickly decreased after 40 cycles (Figure 5e). Meanwhile, in cycles 1 and 50, Cu foam exhibited overpotentials of 81.2 and 48.3 mV, which were much higher than those of TpPa‐Cu (37.2 and 20.2 mV), suggesting a smaller lithium deposition barrier on the surface of TpPa‐Cu (Figure 5f). This may be attributed to the lower work function of the TpPa layer formed in situ, resulting in a small energy barrier for Li‐ion injection, while copper has a work function of up to 4.65 eV. A test was conducted on a full battery assembled with LiFePO_4_ (LFP) cathodes of different mass loads. When the LFP load was 16.5 mg cm^−2^, the discharge capacity of the TpPa‐Li ||LFP battery after 300 cycles was approximately twice that of the pure Li|LFP battery after 100 cycles. These test results reveal that TpPa with low work function and high Young's modulus can be used in actual LMBs. Similarly, Chen et al. prepared in situ thin (10 nm) and rigid (6.8 GPa) COF films as artificial SEI layers of Li anodes for reestablishing lithium plating/stripping and inhibiting Li dendrite growth.^[^ ^71^ ^]^ The sufficient microcellular structure in COF can modulate Li^+^ flow, making the plating/stripping process more uniform. The rigid COF with sufficient micropores effectively reduced the side reaction between electrolyte and Li metal by selectively binding Li^+^. In addition, the COF layer ensured uniform deposition of Li^+^ and played a strong shielding role. AFM result revealed that the PVDF film was broken down when the force increased to 586 nN. However, when the compression force was increased to 2.3 µN, the COF film remained intact. The results indicate that COF films have satisfactory mechanical properties and a wide elastic deformation range, indicating that COF films have good puncture resistance. Subsequently, the symmetric battery cycled at 1.0 mA cm^−2^@1 mAh cm^−2^. For the Li||Li symmetric battery, the electroplating/stripping overpotential gradually increased from the initial 32 mV (vs Li^+^/Li) to 63 mV, and a large voltage fluctuation finally occurred after 100 cycles. This fluctuation might be due to repeated accumulation and destruction of the natural solid–state electrolyte interface (NSEI) layer. In contrast, the COF‐Li symmetrical battery exhibited a stable cycle of steady voltage for 400 h, which may result from a stabilized artificial SEI and a regular Li‐ion flux guided by the COF layer. The Li/S battery suddenly lay off after the 111th cycle due to an internal short circuit. In contrast, the COF‐Li/S battery stably ran for 300 cycles under the same conditions without short‐circuiting. The results show that the LMAs with thin COF membrane as artificial SEI have reliable safety through both hard (mechanical inhibition of Li dendrites) and soft (reconstruction Li^+^ plating/stripping) methods.

#### 3.1.2 COF‐Based Films with Introduction of Groups or Ions

**Figure 6:**
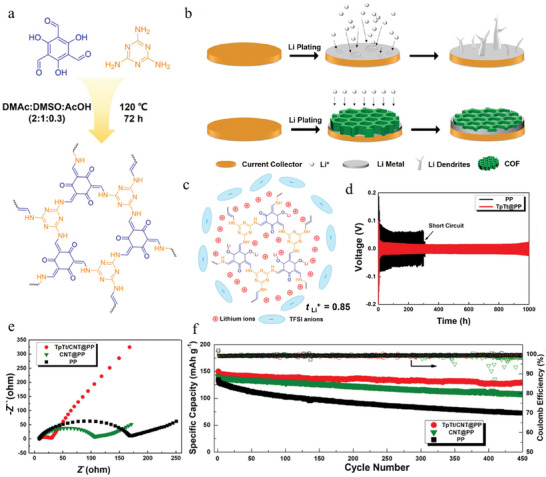
a) The schematic illustration of the β‐ketoenamine‐linked polymerization reaction to produce crystalline TpTt. b) Schematic illustration of LMAs with and without COFs protected interlayer. c) Schematic illustration of the positive charged TpTt. d) The cycling performance of symmetric lithium batteries with and without TpTt coated separators at current density of 1.0 mA cm^−2^. e) The Nyquist plots of Li−LFP batteries with TpTt/CNT@PP, CNT@PP, and PP separators after cycling. f) The long‐term cycling performances of Li−LFP batteries with TpTt/CNT@PP, CNT@PP, and PP separators at 0.5 C. Reproduced with permission.^[^ ^136^ ^]^ Copyright 2021, American Chemical Society.

Uneven Li deposition leads to the discretional development of the branched Li metal to form Li dendrites, which can lead to a low Li utilization rate, fast capacity decay, and poor cycling performance.^[^ ^133^ ^]^ Uniform deposition of Li^+^ can be induced by introducing negative electron groups into COF films as artificial SEI of LMAs, thus inhibiting dendrite growth.^[^ ^70^ ^]^ The fabrication of artificial SEI can mitigate traditional lithium protection limitations and adjust surface properties.^[^ ^134^, ^135^ ^]^ Li et al. used 2, 4, 6‐trihydroxybenzene‐1,3,5‐trietaldehyde and melamine as raw materials to synthesize a polar carbonyl polymer skeleton (COF‐TpTt) with keto‐enol tautomerism and triazine ring arrangement as a protective layer of LMAs using the Schieff reduction method (**Figure** **6**a).^[^ ^136^ ^]^ Through a pre‐designed synthesis strategy, the COF‐TpTt exhibited a 2D planar porous structure with periodic subunit arrangement and expansion. The triazine ring rich in lone pair electrons acted as the donor to attract Li^+^, and the carbonyl group effectively served as the Li^+^ anchor site to coordinate Li^+^. These periodically arranged subunits markedly induce the uniform distribution of Li^+^ flux to ensure the smooth deposition of Li‐ion and the formation of fewer Li dendrites. Within the framework, the substantial triazine rings strongly acted as electron‐rich sites to attract Li‐ion, while carbonyl groups with high dielectric constants acted as polar Li anchoring positions. These well‐ordered subunits greatly enhance the ionization of electrolyte molecules and induce the even distribution of Li‐ion fluxes within the porous polymer skeleton (Figure 6b). The compact arrangement of polar groups can create considerable active sites to maximize Li affinity and anchoring ability (Figure 6c). Therefore, TpTt‐modified cells can effectively inhibit Li dendrite and enhance working performance. In a half cell, a symmetrical cell with a TpTt protective layer can significantly achieve long‐term cycle stability of 1000 h below 1.0 mA cm^−2^ (Figure 6d). In contrast, symmetrical batteries without a TpTt protective layer (Li||Li) suffer from severely large voltage fluctuations or even short circuits due to severe polarization during cycling and the dendritic Li tip piercing the partition. This phenomenon confirms that TpTt can accurately guide the Li‐ion flow and effectively inhibit the Li dendrites during the long‐term cyclic process, thus weakening the dynamic barrier of Li homogeneous nucleation. In the full battery, the interface resistance of the battery using TpTt/CNT was reduced, and ion transport was accelerated compared with the battery using CNT and the bare Li (Figure 6e). Batteries assembled with the TpTt/CNT exhibited the lowest capacity loss at more than 450 cycles at 0.5C (Figure 6f). The excellent cycling properties indicate that the Li‐philic TpTt can ensure uniform Li^+^ flux regulation. Similar to the triazine ring, highly electronegative fluoride ions can also induce rapid Li^+^ migration. Yang et al. dispersed 2,5‐bis((3,3,4,4,5,5,6,6,6‐nonafluorohexyl)oxy) terephthalohydrazide and 1,3,5‐trisformylbenzene (Tf) into a mixture of 1, 4‐dioxane, tritoluene and acetic acid catalysts to form a perfluoroalkyl functionalized COFs (COF‐F6) as an artificial SEI with high stability and super proton conductivity to stabilize LMAs. At the same time, COF42 was prepared by a similar method.^[^ ^137^ ^]^ The binding energy between solvents, COF‐F6, COF42, TFSI^−^, and Li^+^ was calculated using DFT. The result showed that the binding energy between COF‐F6 and Li^+^ was the largest. Due to the large adsorption energy of COF‐F6, more Li^+^ can be captured from the LiTFSI and released, and finally realize the dissociation of LiTFSI. Additionally, the fluorine with high electronegativity can regulate the local electron cloud density to ensure the smooth transport of Li‐ion flux. The above effect increases the transfer number of Li^+^ (0.7), inhibiting Li dendrites. Both experimental results and DFT calculations confirm that perfluoroalkyl can accelerate Li‐ion transfer, hinder TFSI^−^ migration, and inhibit lithium dendrites. Therefore, symmetric batteries protected by COF‐F6 can be stably cycled over 5500 h at 2 mA cm^−2^@2 mAh cm^−2^, while symmetric batteries using COF42 and bare Li can both experience short circuit failure within cycles of less than 1000 h. The full batteries using COF‐F6 exhibited a significant capacity retention rate and CE of ≈100 after 500 cycles at 1C. In contrast, COF42 exhibited significant capacity and CE decay at ≈250 cycles after it experienced short circuit failure. Moreover, the pure Li showed a significant capacity decline at ≈80 cycles, and the cycle life remained at ≈200 cycles. These results show the functionalized COF‐F6 greatly improves the cycling performance of LMBs and provides a new path for inhibiting the growth of Li dendrites.

### 3.2 ZIBs

Similar to Li, Zn metal anodes also face severe dendrite growth problems. Zn can easily penetrate the diaphragm owing to its higher hardness, leading to an internal short circuit, which exacerbates the impact of Zn dendrite growth on ZIBs. In the process of Zn deposition, Zn^2+^ on the anode surface are reduced to Zn and freely diffused on the Zn electrode surface. This unrestricted diffusion causes Zn atoms to accumulate or locate in places of high energy (protrusions, boundaries, lattice defects, and impurities) to form initial Zn crystal nuclei.^[^ ^138^ ^]^ The uneven deposition of Zn atoms aggravates the non‐uniform distribution of the electric field, which in turn affects the subsequent metal electrodeposition.^[^ ^139^ ^]^ As a result, subsequent charge carriers prefer to decrease at the existing tip to minimize surface energy, leading to tip growth and eventual dendrite formation. In addition, Zn has a hexagonal, tightly packed crystal structure. Zn atoms are arranged in a hexagonal shape, and Zn crystal grows in the direction of {001} crystal plane. Vertically grown sheets are more likely to form large dendrites, while horizontally formed sheets often inhibit dendrite formation.^[^ ^140^, ^141^ ^]^ Therefore, the morphology of the electrodeposition of material onto a substrate is closely related to crystal orientation. Thus, the uncontrolled 2D diffusion behavior, non‐uniform electric field distribution, and the growth state of zinc metal are the main causes of dendrite formation. The growth of Zn dendrites exacerbates the side reactions (i.e., corrosion and HER) by expanding the exposed Zn anode area.^[^ ^142^ ^]^ Among them, the appearance of HER is directly related to the thermodynamic instability of Zn in a water medium, and it is a strong competitive reaction for reducing active Zn.^[^ ^143^ ^]^ Especially in alkaline electrolytes, the higher the reduction potential, the easier the reduction. The standard reduction potential of Zn/ZnO redox pairs is −1.26 V (vs SHE), and HER potential is −0.83 V (vs SHE). As a result, HER will occur first (Equations 1 and 2).^[^ ^144^ ^]^ Therefore, alkaline Zn‐based cells exhibit a serious HER problem, which restrains their practical application. Similarly, in the weakly acidic media, the standard reduction potential of Zn/Zn^2+^ fall behind the hydrogen evolution potential (0 V vs SHE), resulting in prior HER (Equation 3). In contrast, in the neutral electrolyte, the standard reduction potential of Zn/Zn^2+^ (−0.76 V vs SHE) is higher than the value of hydrogen evolution, indicating that HER does not thermodynamically advance the reduction of Zn^2+^ (Equation 4). However, the electrolyte cannot easily remain neutral after hydrolysis because of the polyvalent ionic nature of Zn^2+^. Thus, Zn^2+^ will be present in the form of (Zn(H_2_O)_6_)^2+^, resulting in slow kinetics.^[^ ^139^, ^145^ ^]^ In addition, the dissolution of Zn^2+^ can increase interfacial polarization, thus increasing charge transfer impedance and enhancing hydrogen evolution. The hydrogen generated will aggravate the internal pressure of the battery, thereby causing volume expansion and even explosion of the battery. HER also changes the local pH, generates more OH^−^, and produces some undissolved side‐products that can inactivate the anode.^[^ ^146^ ^]^ Taking the extensively used ZnSO_4_ electrolyte as an example, the main side‐product is ZnSO_4_[Zn(OH)_2_]_3_·xH_2_O (Equation 5). ZnSO_4_[Zn(OH)_2_]_3_·xH_2_O is ineffectively in separating the electrolyte from the fresh Zn, causing persistent corrosion of the anode and forming inert side‐products on the Zn surface, resulting in a passivation layer. Corrosion and passivation can lead to uneven surfaces, resulting in non‐uniform deposition of Zn and the growth of dendrites.^[^ ^147^ ^]^ Therefore, a vicious corrosion cycle, passivation – dendrite growth – HER will appear on the Zn anode surface.

(1) $$\mathrm{ZnO}+2e^{-}+H_{2}O↔\mathrm{Zn}+2{\mathrm{OH}}^{-}\left(-1.26V\right)$$

(2) $$2H_{2}O+2e^{-}↔H_{2}↑+2{\mathrm{OH}}^{-}\left(-0.83V\right)$$

(3) $${\mathrm{Zn}}^{2+}+2e^{-}↔\mathrm{Zn}\left(-0.76V\right)$$

(4) $$2H^{+}+2e^{-}↔H_{2}↑\left(0V\right)$$

(5) $$\begin{array}{c} 3{\mathrm{Zn}}^{2+}+6{\mathrm{OH}}^{-}+{\mathrm{ZnSO}}_{4}+{\mathrm{xH}}_{2}O↔{\mathrm{ZnSO}}_{4}{\left[\mathrm{Zn}{\left(\mathrm{OH}\right)}_{2}\right]}_{3}\cdot {\mathrm{xH}}_{2}O \end{array}$$

Because of these problems, researchers have focused their attention on COFs, which are used to solve similar problems in LMBs, yielding remarkable results. COFs have the characteristics of designable structures, which allows the addition of polar functional groups with high interaction activity with Zn^2+^, water molecules, and Zn surfaces during the design process to promote the formation of a zincophilic layer. The large surface area of COFs can decrease the local current density and effectively regulate the distribution of Zn^2+^ flow and spatial electric field, thus speeding zinc deposition kinetics and impeding H_2_ formation.^[^ ^147^ ^]^ The advantages of COF materials with enhanced electrochemical properties make their applications successful in energy storage and electrochemistry. Therefore, in this section, we briefly describe some successful cases of COFs used to solve the Zn anode problem.

As ZIBs cycle times increase, the uncontrolled growth of Zn anode dendrites and the uneven deposition of Zn inevitably lead to changes in the volume of ZIBs. The use of high‐modulus film can inhibit dendrite piercing, while the use of low‐modulus and high‐elastic protective film can tolerate a certain amount of volume change within the battery without rupturing or allowing dendrite piercing, which may be another solution. Park et al. prepared ultra‐thin (20–100 nm), strong, and flexible (<200 MPa) COF films (DIP‐D‐COF) using aldehyde precursors 1, 3, 5‐triphloroglucinol (TFP) and diaminoquinone (DAAQ) amino linking agents as Zn anode protective films.^[^ ^148^ ^]^ The electron‐rich ketones and imine functional groups in DIP‐D‐COF had a strong affinity for Zn^2+^, facilitated interaction with Zn^2+^, and prompted the transport of the ions through the pore wall via a point‐to‐point jumping mechanism.^[^ ^149^ ^]^ In addition, compared with bare Zn, DIP‐D‐COF had a lower Zn nucleation overpotential, which had a lower Zn deposition energy barrier, making DIP‐D‐COF an advantageous material for initial Zn deposition. Therefore, DIP‐D‐COF can promote the even deposition of Zn^2+^, thus inhibiting the formation and growth of Zn dendrites. The DIP‐D‐COF surface did not show any significant cracks or other deterioration after 200 fold/recovery cycles, and the capacity remained the same when the battery was bent, twisted, crumpled, or rolled. The power supply was normal even in the folded state. The results show that DIP‐D‐COF is highly flexible and can withstand the volume change in the battery during the working process. Consequently, in a symmetric cell, the DIP‐D‐COF coated Zn electrode can cycle stably for more than 300 h without significant voltage fluctuation at 1 mA cm^−2^@1 mAh cm^−2^. The bare Zn electrode showed sudden voltage fluctuations and an irreversible rise in polarization voltage at ≈14 h, resulting in a short circuit. The test conducted on a full battery assembled with a δ‐MnO_2_ cathode and a bare Zn or DIP‐D‐COF@Zn anode showed that DIP‐D@Zn||δ‐MnO_2_ achieved a good performance at 2 A g^−1^, with CE greater than 99.9% and capacity retention of 88.5% after 300 cycles. By contrast, Zn||δ‐MnO_2_ showed a significant decrease in capacity, and the retention rate was only 58.7%. The battery is often accompanied by electrolyte consumption or spontaneous side reactions during its operation. The by‐products produced by the side reaction (H_2_ and Zn_4_SO_4_(OH)_6_·xH_2_O) inevitably reduce the zinc utilization rate and cycle life and increase the contact area between the zinc anode and electrolyte, resulting in a rapid increase in the Zn^2+^ flux and ultimately the Zn dendrites formation. Additionally, when zinc deposition on the anode is uneven, a strong concentrated electric field will be formed in the concentrated deposition area of Zn, leading to more vigorous hydrogen evolution. Excessive H_2_ precipitation may rapidly increase the internal pressure or rapidly consume the electrolyte of the airtight battery, directly resulting in an early short circuit of the battery and even posing safety issues (such as electrolyte leakage and explosion). Therefore, it is crucial to address the root cause of HER problem.

**Figure 7:**
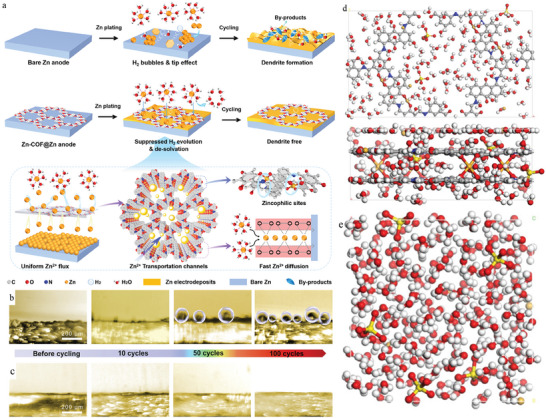
a) The structure design and mechanism elucidation of Zn‐COF@Zn anode. In situ operando optical microscope images of b) bare Zn and c) PVC‐Zn‐AAn‐COF@Zn during plating/stripping at 20 mA cm^−2^. The snapshot of molecule dynamics simulations of d) Zn‐AAn‐COF@Zn (top view and side view) and e) bare Zn. Reproduced with permission.^[^ ^150^ ^]^ Copyright 2022, Wiley‐VCH GmbH.

To better explain the effect of different components on inhibiting HER and dendrite growth, Guo et al. directly metalized COFs with Zn(CH_3_COO)_2_ using a hydrothermal method and synthesized Zn‐AAn‐COF, Zn‐DAA‐COF, and Zn‐DAAQ‐COF as model platforms to control Zn^2+^ flux and H_2_ evolution in ZABs (**Figure** **7**a).^[^ ^150^ ^]^ Zn‐AAn‐COF, with its zincophilic groups (such as C═O and C═N), high porosity, and numerous Zn nucleation sites, is an ideal model platform for ZABs research. They theoretically and experimentally demonstrated that Zn‐AAn‐COF inhibited HER on the electrode. They fabricated electrodes with COF protection by casting a COF‐based polyvinyl chloride (PVC) suspension on the zinc plate (PVC‐Zn‐AAn‐COF@Zn). Then, the plating/stripping behavior of Zn in transparent symmetric cells (20 mA cm^−2^@1 mAh cm^−2^) was monitored by in situ optical microscopy. The nucleations and protrusions were observed on the zinc foil after 10 cycles and gradually changed into zinc dendrites with the number of cycles. After 100 cycles, extremely disordered Zn deposition, substantial H_2_ bubbles, and serious corrosion were observed on the Zn foil surface (Figure 7b). In contrast, after 100 cycles under the same conditions, uniform dense Zn deposits were observed on the PVC‐Zn‐AAn‐COF@Zn foil with no signs of H_2_ bubbles and dendrite growth (Figure 7c). Theoretically, the mechanism of inhibiting H_2_ precipitation was investigated by calculating the Gibbs free energy (ΔG_H*_) of bare Zn and Zn‐COF@Zn adsorbed to H^*^. H_2_ was produced by inert hydrolytic dissociation on the sample surface to generate adsorbed H^*^ and OH^*^. The calculated results show that the ΔG_H*_ value of Zn‐AAn‐COF@Zn (2.94 eV) is six times that of bare Zn (0.52 eV), showing that H^*^ adsorption capacity of Zn‐AAn‐COF@Zn is weak. The hydrolysis dissociation process of Zn‐AAn‐COF@Zn anodes was more difficult than that of Zn‐DAA‐COF@Zn and Zn‐DAAQ‐COF@Zn anodes, thus supplying less proton, which effectively inhibits H_2_ precipitation. In addition, the coordination number of Zn‐H_2_O on the anode surface was further calculated through molecular dynamics simulation, and the results showed that the coordination number of Zn‐AAn‐COF (1) was significantly lower than that of bare Zn (6), suggesting that Zn‐AAn‐COF was more effective to removing hydrated molecules on the zinc surface (Figure 7d,e). Desolvation hinders the formation of by‐products and competitive H_2_.^[^ ^151^ ^]^ Subsequently, in the full battery test, the PVC‐Zn‐AAn‐COF@Zn||MnO_2_ cell at 2000 mA g^−1^ maintained a high capacity of 90.9 mAh g^−1^ and a CE of 100%, corresponding to a capacity retention rate of 76.2% after 1000 cycles. In contrast, Zn||MnO_2_ batteries were only stable for the first few cycles, after which the specific capacity quickly decayed. In addition, the PVC‐Zn‐AAn‐COF@Zn||MnO_2_ battery bag powered light‐emitting diode indicators, indicating its application prospects in portable electronic devices.

**Figure 8:**
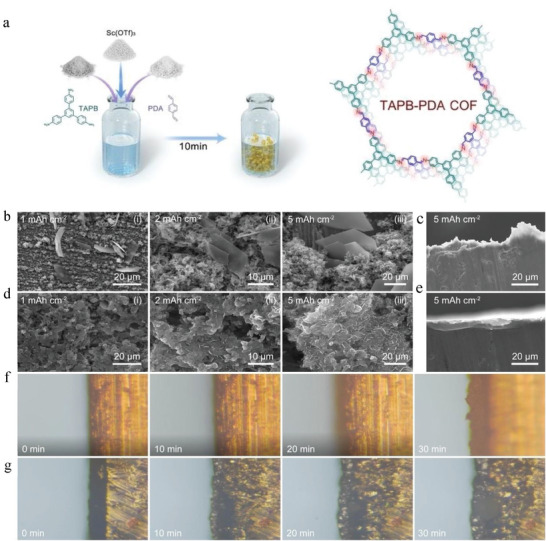
a) Diagram of synthesis method and molecular structure of TAPB‐PDA COF. Investigation of longitudinal Zn growth mode: ex situ scanning electron microscopy (SEM) images of b) bare Zn and d) Zn@COF at different deposition capacities of 1, 2, and 5 mAh cm^−2^; cross section SEM images of c) bare Zn and e) Zn@COF after depositing for 5 mAh cm^−2^. In situ optical microscope images of f) bare Zn and g) Zn@COF during the real‐time observation of 30 min. Reproduced with permission.^[^ ^105^ ^]^ Copyright 2023, Wiley‐VCH GmbH.

Most current studies on ZIBs have focused on strategies to inhibit the growth of dendrites, ignoring other challenges related to the practical application of ZIBs, such as achieving large area capacity or high discharge depth (DOD). The current dendrite protection method mainly focuses on the Zn deposition behavior at the anode/electrolyte interface. The thin (<1 µm) Zn deposition is a method used to inhibit the dendrite growth; however, it is limited by the problem of low capacity (<0.585 mAh cm^−2^). Specifically, Zn deposition is mainly conducted in a relatively uniform manner at the low surface–volume interface. The growth of Zn in outer space with high surface volume is prone to random dendrite growth.^[^ ^105^, ^152^ ^]^ Therefore, achieving high area capacity and uniform Zn deposition at the same time is difficult. Zhi et al. recently proposed and used acetaldehyde and aniline monomers as raw materials to synthesize TAPB‐PDA COF as an intermediate layer through a solvothermal condensation reaction at room temperature.^[^ ^105^ ^]^ The synthesized TAPB‐PDA COF controlled the longitudinal growth of Zn, increased the capacity, and inhibited dendrite growth to a certain extent (**Figure** **8**a). As shown in Figure 8b, at a low surface capacity of 1 mAh cm^−2^, a small number of deposited Zn clusters and sparsely distributed basic Zn sulfate appear on bare zinc anode. As the capacity increased, the Zn dendrites became larger to ≈20 µm in size, vertically grew on the Zn anode, and showed significant anisotropy along the zinc anode surface (Figure 8c). Contrastively, COF@Zn anodes always maintained a smooth surface during a gradual increase in capacity from 1 to 5 mAh cm^−2^ (Figure 8d). Even under high surface volume conditions, Zn deposition was only observed on the surface of the COF layer. No large size Zn plate was observed, making the surface uniform (Figure 8e). As time prolonged, noticeable Zn dendrites appeared on bare zinc anode (Figure 8f). Contrastively, Zn@COF anodes maintained a smooth surface throughout the deposition/growth process (Figure 8g). As uniform zinc plating/stripping proceeds, the contact between the battery components becomes closer, thus gradually reducing the impedance.^[^ ^153^ ^]^ Therefore, the uniform longitudinal growth of Zn is attributed to the electrostatic and chemical interactions caused by the N‐rich COF. In addition, the hydrophobicity of COF (141.33°) was much higher than that of Zn metal (57.96°). Therefore, the hydrophobic intermediate layer may promote the dissolution of hydrated Zn^2+^ in the electrolyte and inhibit parasitic reactions. Then, the effect of COF on Zn deposition stability was tested. In the whole current density range, the Zn@COF symmetric battery exhibited lower voltage lag and smaller Zn nucleation overpotential than the bare Zn‐symmetric battery, indicating that COF modification promoted the Zn nucleation process. In terms of cycle stability, the bare Zn‐symmetric battery was short‐circuited after 154 h when tested at 5 mA cm^−2^, while the Zn@COF symmetric cell was stably operated after 1000 h, indicating its life was extended more than five times. In the case of I_2_ loading masses of 1.95 mg cm^−2^ and 1 A g^−1^, long‐term cycling tests show that Zn@COF||AC+I_2_ batteries have satisfactory cycle stability and maintain a high capacity retention rate and CE (≈100%) after 10000 cycles, which is much better than that of bare Zn. Based on the above results, COF can regulate the longitudinal uniform growth of Zn, thereby improving the cycle and rate performances of Zn‐I_2_ batteries.

## 4 Improvement Strategies for COFs as Protective Films for Li and Zn Metal Anode

The introduction of COFs into rechargeable batteries as a protective film for the metal anode has minimized the problems faced by the anode and somewhat improved the battery performance. However, some open problems are still encountered when using some of the COFs as anode protection film. For example, some of the COFs have insufficient active site exposure, insufficient affinity for metal ions (Li^+^, Zn^2+^), and underutilized metal ion induction, thus severely affecting the ion transport and deposition process, thereby limiting the battery cycle efficiency. Specifically, during battery operation, electrons migrate through the external circuit when the electrode undergoes an electrochemical redox reaction, and metal cations shuttle between the anode and cathode in the electrolyte, forming a closed loop.^[^ ^154^ ^]^ Both electrons and metal cation migrations play pivotal roles in achieving high‐speed electrochemical performances. Ion conduction, in particular, is present in every vital component of the battery, such as the electrolyte, separator, cathode and anode. The potential equation of ion conduction behavior (Equation 6) shows that apart from the open‐circuit voltage E_0_ determined by the difference between the standard cathode potential and the anode potential, other parameters are affected by the ion conduction behavior.^[^ ^155^ ^]^ Moreover, due to the polarization and internal resistance, the open‐circuit voltage of the cell is higher than the effective output voltage, indicating voltage loss is significantly reduced in energy density.^[^ ^154^ ^]^ Therefore, optimizing the COF protective films for Li and Zn anodes from the aspect of ion conduction is vital for producing practical secondary batteries. Based on the above challenges, we can achieve a better solution by modulating COFs or introducing some favorable groups for optimization. In this section, we detail some optimization methods.

(6) E=E0−[(ηct)a+(ηct)c]−[(ηc)a+(ηc)c]−IRi

E: working voltage, E_0_: open‐circuit voltage, (η_ct_)_a_: charge transfer overvoltage at the anode sides, (η_ct_)_c_: charge transfer overpotential at the cathode sides, (η_c_)_a_: concentration polarizations at the anode surfaces, (η_c_)_c_ concentration polarizations at the cathode surfaces, I: working current, R_i_: battery internal resistance.

### 4.1 Aperture Pore Size Adjustment

**Figure 9:**
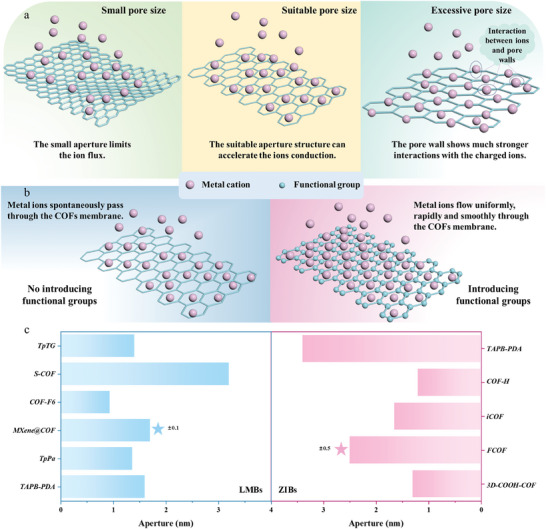
Comparison of a) COF aperture and b) wall optimization. c) The pore sizes of COF protective films on Li and Zn anodes.

**Figure 10:**
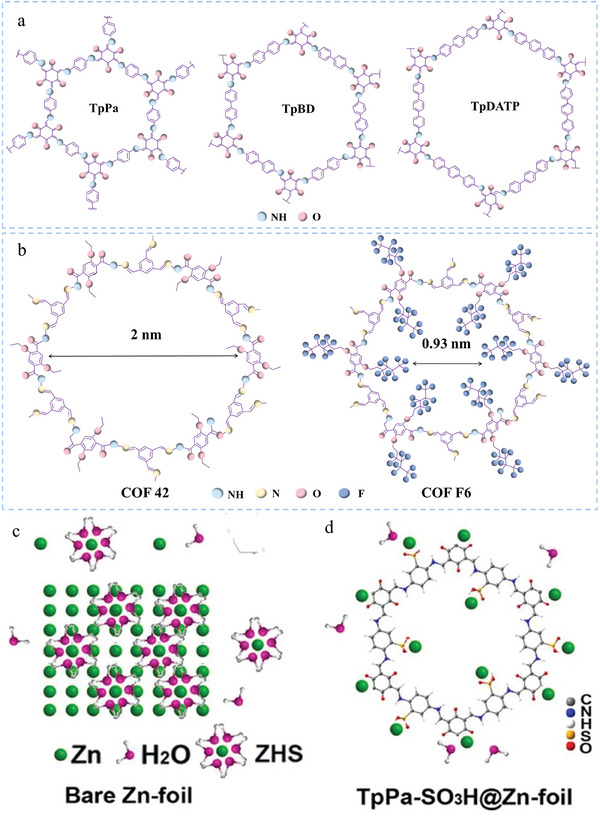
Schematic structure of TpPa‐COF, TpBD‐COF, and TpDATP‐COF a) and COF 42, COF‐F6 b). Schematic illustrations of the desolvation process of the Zn^2+^ on bare Zn foil c) and TpPa‐SO_3_H@Zn‐foil d). Reproduced with permission.^[^ ^107^ ^]^ Copyright 2022, Elsevier B.V.

COFs are used as metal anode protective film in rechargeable batteries, and their long‐range ordered nanochannels provide a conductive pathway for metal ions, which is conducive to the charge and discharge dynamics of rechargeable batteries. The aperture size of the COF can be adjusted over a wide range by precisely designing the length and size of the building blocks (**Figure** **9**a).^[^ ^156^ ^]^ The diameters of Li^+^ and Zn^2+^ are significantly smaller than the minimum pore diameter of COFs.^[^ ^126^ ^]^ Therefore, when COFs are used to improve metal anodes, researchers do not pay much attention to the adjustment of COF pore size. As a result, they ignore the effect of its pore size on battery performance. Currently, COFs for rechargeable batteries have aperture sizes between 0.56 and 5.06 nm.^[^ ^156^ ^]^ For example, a highly stable COF (COF‐H) with a flower‐like structure and abundant alkynyl units, synthesized by Hu et al. in the AA‐stacking mode, was used to achieve an artificial protective layer on Zn anode with a pore size of 1.2 nm.^[^ ^157^ ^]^ Through an in situ Schiff base reaction, Chen et al. prepared a microporous organic polymer (COF_TAPB‐PDA_) with robust covalent bonds and a pore size of 1.5 nm as a protective film for Li metal anode to inhibit dendrite growth.^[^ ^71^ ^]^ Guo et al. synthesized a COF film on MXene (Ti_3_C_2_T_x_) nanosheets through Schiff base condensation between monomer aldehyde and amino group with help of an acetic acid catalyst and applied it in Li metal batteries as Li‐ion carrier.^[^ ^158^ ^]^ The non‐local DFT model reveals that the micropores with pore size distribution of ≈1.6–1.8 nm are in good agreement with the COF‐LZU1 pore. Meanwhile, mesoporous pores of 2–40 nm were obtained through the COF intercalation between MXene nanosheets. In general, the pore sizes of COFs as Li and Zn metal anode protective membranes are basically microporous and mesoporous, and almost intermediate between 0.9–3.5 nm (Figure 9c). From the perspective of the battery working principle, the size of the COFs aperture during charging and discharging will directly affect the flux of plating / stripping of the metal ions at the metal anode. From the aspect of metal anode protection, too small pore size may lead to lower ion flux, which leads to low metal anode utilization. Excessive pore size of the ion flux leads to uneven ion deposition. Therefore, the proper pore size of COFs films is crucial for protecting the metal anode. However, a direct relationship between ionic conductivity and pore size distribution is difficult to establish due to the complex ionic conductive environment and a wide variety of COFs. In general, the larger the pore size of COFs, the larger the allowable ion flux and the faster the ion transport. However, Zhao et al. synthesized three different COFs (1,3,5‐triformylphloroglucinol (Tp) p‐phenylenediamine (Pa)‐COF (TpPa‐COF), Tp 4,4‐diamino‐p‐terphenyl (DATP)‐COF (TpDATP‐COF), and Tp benzidine (BD)‐COF (TpBD‐COF)) (**Figure** **10**a).^[^ ^159^ ^]^ The pore size of TpPa‐COF is the smallest among the three COFs. However, it had a low interfacial impedence when it was deposited on Li metal surfaces. Therefore, compared with TpBD‐COF and TpDATP‐COF membranes, TpPa‐COF membranes achieved faster Li^+^ transport mechanics. In addition, in COF42 (2.0 nm) and COF‐F6 (0.93 nm) prepared by Yang et al., COF‐F6 with the small aperture corresponded to low impedance and high ion transfer numbers, whereas large‐pore COF‐F6 corresponded to high impedance and low ion transfer numbers (Figure 10b).^[^ ^137^ ^]^ It can be suggested that the pore size is not the only factor affecting ionic conductivity. The large pores indeed provide more free volumes for ion migration and have the potential to achieve fast conduction rates. However, because of the stronger interaction between the large pore wall corresponding to the large aperture and the charged ions, the ion flux and transport rate are greatly reduced. Thus, larger pore sizes do not necessarily favor ion transport. In contrast, small pores are more conducive to dissociating salts, producing abundant mobile single ions and achieving rapid ion conduction. Therefore, to regulate ion conduction behavior in secondary metal cells, the design principles should be based on pore size and the interaction between pore walls and moving ions.

### 4.2 Pore Wall Decoration

The influence of pore wall decoration on the ion transport is much greater than the adjustment of pore size in COFs (Figure 9b). As a routine approach to expand functions of COFs, the pore wall modification method can be mainly achieved through two methods: precise pre‐design of functional groups in COF monomers and the introduction of side group functions into the COF skeleton through synthetic methods.^[^ ^160^ ^]^ The first method is considered an effective and straightforward strategy for building COFs. The functional groups in target COFs synthesized by this method are uniformly distributed and adjustable.^[^ ^161^ ^]^ For example, Zhao et al. developed an interface reaction strategy using 2,4,6‐triformylphroglucinol (Tp) and pre‐designed 2,5‐diaminobenzenesulfonic acid (Pa‐SO_3_H) containing ‐SO_3_H on p‐phenylenediamine to synthesize TpPa‐SO_3_H COF as a protective film for zinc anodes in ZIBs (Figures 10c,d).^[^ ^107^ ^]^ Compared with TpPa@Zn‐foil (5.25 mS cm^−1^) and bare Zn foil (3.86 mS cm^−1^), batteries assembled with TpPa‐SO_3_H@Zn‐foil showed a higher ionic conductivity (10.48 mS cm^−1^) after cycling owing to the special coordination configuration between Zn^2+^ and TpPa‐SO_3_H membrane.^[^ ^162^ ^]^ In addition, the ‐SO_3_H endowed TpPa‐SO_3_H film with good affinity to the zinc anode, thus improving the conductivity of Zn ions.^[^ ^163^ ^]^ Therefore, numerous negatively charged polar sulfonic acid groups in the TpPa‐SO_3_H membrane endowed the film with excellent zincophilic properties in aqueous solutions. Moreover, the pores of the TpPa‐SO_3_H film can act as nanoscale Zn^2+^ reservoirs, facilitating faster ion transport and lower charge transfer resistance between the TpPa‐SO_3_H membrane and the zinc foil. During the galvanizing/stripping process, each Zn^2+^ was ligated with six H_2_O molecules, resulting in a high dissolution energy of Zn^2+^.^[^ ^164^ ^]^ TpPa‐SO_3_H membrane can reduce the dissolution energy by releasing a large amount of H^+^ and establishing a dynamic balance with OH^−^, thus restraining the formation of side‐products. In addition, the sulfonic acid group in TpPa‐SO_3_H film can synergize with Zn^2+^ to increase the zincophilic of the anode, facilitating the formation of uniform Zn nucleation and deposition.^[^ ^106^ ^]^ These properties of TpPa‐SO_3_H can effectively enhance the rate capacity and cycle stability of batteries. Hence, compared with bare zinc foil and TpPa@Zn‐foil, the TpPa‐SO_3_H@Zn‐foil anode had clear Zn^2+^ channels and low Zn nucleation overpotential. The sulfonic acid group can coordinate with zinc ion, reduce the dissolution barrier of Zn^2+^, form rich Zn transport channels, and inhibit the random diffusion of Zn ions. The zincophilic property of TpPa‐SO_3_H can promote the rapid transport of Zn^2+^, regulate the dissolution of Zn(H_2_O)_6_^2+^, and inhibit dendrite growth and secondary reactions. Therefore, COFs with functional groups introduced by pre‐design are more efficient in solving the Zn anode problem than the original COFs. However, the types of functional groups that can be used to satisfy the structural law without affecting the synthesis of COFs are limited.^[^ ^165^ ^]^ Therefore, the post‐synthetic modification method has become a popular strategy for modifying the pore wall owing to its nondestructive effect on the crystal structure of COFs. This method can be further used to modify established COFs through covalent connections between functional side chains and existing overhanging groups, such as ─OH, ─NH_2_,═CH_2_, and ─CN_3_. Based on different transport mechanisms, COFs with anionic, neutral, or cationic frameworks can be adjusted according to the pore environment of COFs to accelerate ion conduction. The interaction between the modified pore wall and the ion can dissociate the salt and produce free cations, thus facilitating rapid transportation.^[^ ^136^ ^]^ Kang et al. used imide bonding to form COF‐LZU1 containing aldehyde functional groups as a protective film for the Li anode.^[^ ^166^ ^]^ The interaction between the aldehyde functional group in COF‐LZU1 and the bis (trifluoromethanesulfonyl) imide anion (TFSI^−^) in the electrolyte can fix the anion, which is conducive to reducing the space charge effect of the anode. The imine bond exhibited suitable electrolyte wettability and Li affinity, promoting uniform Li‐ion flux and resulting in low voltage hysteresis. Based on the above characteristics, Li||Cu batteries assembled with COF‐LZU1 achieved a excellent CE of >99%, and Li–Li batteries achieved a lifetime of over 2000 h with potential hysteresis of only 35 mV. Guo et al. prepared two different hydrazine‐linked 2D COFs (COF42 and COF‐F6, Figure 10b) as artificial SEI to stabilize LMAs.^[^ ^137^ ^]^ After the perfluoroalkyl functionalization of COF‐F6, the dissociation of LiTFSI and the dissolution of Li^+^ were more easily realized under the competition of electronegative fluoride ion (F^−^) and TFSI^−^. Moreover, the influence of fluorine repulsion hindered the migration of TFSI^−^ and significantly improved t_Li_^+^, inhibiting the formation of Li dendrites. Compared with bare Li and COF42@Li, COF‐F6@Li exhibited excellent electrochemical properties, such as lower polarization voltage, higher Li^+^ transfer number (t_Li_^+^, 0.7), and higher CE in Li||Cu batteries. Therefore, this material and strategy can provide a new perspective for regulating Li metal anode surfaces.

### 4.3 Membrane Thickness Regulation

In general, during COFs growth, the interlayers tend to be tightly arranged in a parallel stacking mode due to the π‐π interaction. However, due to the long transport distance of ions caused by the tight arrangement of the 2D layer, it is difficult for ions to pass through the channel of COFs or penetrate into the active site inside the skeleton. The longer transmission distance inevitably reduces the ion conductivity, thus increasing the interfacial impedance of the cell. Therefore, in order to maximize the effectiveness of protective COF films on metal anode, the thickness of COF film should be an important problem. Some researchers have paid attention to this problem. For example, TaPa‐COF films with a thickness of 80, 180, and 300 nm were prepared as lithium metal anode protective films by Zhao et al.^[^ ^108^ ^]^ The EIS test after precirculation treatment found that the interface impedance of 80, 180, and 300 nm TaPa‐COF films were 42.03, 86.04, and 119.9 Ω, respectively. The subsequent comparison of the voltage distribution of TpPa‐COF films at 1 mAh cm^−2^@1 mA cm^−2^ found that the 80 nm film had the lowest overpotential, which would be more conducive to achieve smooth lithium deposition. In addition, they subsequently explored the effects of FCOF films (100, 250, and 500 nm) in their study as a zinc anode protective layer.^[^ ^165^ ^]^ It was shown that the 100 nm thick film had the lowest interfacial resistance. They concluded that the relatively thin COF protective film will be more conducive to improve the ion conductivity to reduce the interfacial impedance, thus improving the battery performance. According to the existing reports, it seems that the thinner the COF film thickness, the higher the ion conductivity, the lower the interface resistance. However, based on the analysis of the anode and electrolyte properties, the excessive active site exposed by the excessively thin COF protective membrane may absorb excessive irrelevant ions from the electrolyte, thus hindering the transport of metal cations and eventually leading to a decrease in cell capacity. However, this thin COF membrane, which exposes more active sites, seems to be more suitable for the cathode, according to the modification strategy of the cathode. For example, the addition of exCOFs (bulk COF exfoliation) stripped from bulk COFs to the cathode of Zn bromide battery cells and the use of COF as an anode protective layer collaboratively improve the electrochemical performance of cells. After 1000 cycles, the assembled COF‐Zn||Br_2_‐exCOF battery had a capacity retention rate of ≈83% and a CE of ≈99%.^[^ ^167^ ^]^ In conclusion, the effect of membrane thickness on battery performance is determined by many factors, and should be further studied.

In addition, several methods, including crystallinity regulation and guest‐promoting transport, have been developed to optimize COFs. The crystallinity of COFs is considered a significant factor affecting ion conductivity behavior. However, it is difficult to control the crystallinity of COFs because the covalent bond between building blocks is strong, and the invertibility of bond formation is low.^[^ ^154^ ^]^ As a result, only a few studies on the crystallinity regulation of COFs have been recorded. Although COFs have good ion transfer channels, problems, such as strong coulomb interactions between cations and anions and a lack of transport media in COF pores, still hinder ion conduction. Some researchers have proposed accelerating ionic conduction by incorporating ionic liquid (IL) in COF. However, this optimization method is commonly used in COFs for electrolytes. For example, Shan et al. constructed a thiophene‐based imine‐linked COFs as a Li^+^ conductive composite electrolyte by doping an IL in a 1D nanochannel of COFs.^[^ ^168^ ^]^ The COF‐IL composite electrolyte exhibited excellent thermal stability (up to 400 °C) and extremely high Li^+^ conductivity (2.60 × 10^−3^ S cm^−1^). This work provides insight into new avenues for developing fast Li^+^ conductive COF‐based electrolytes for high‐temperature solid–state LIBs.

## 5 Summary and Outlook

**Figure 11:**
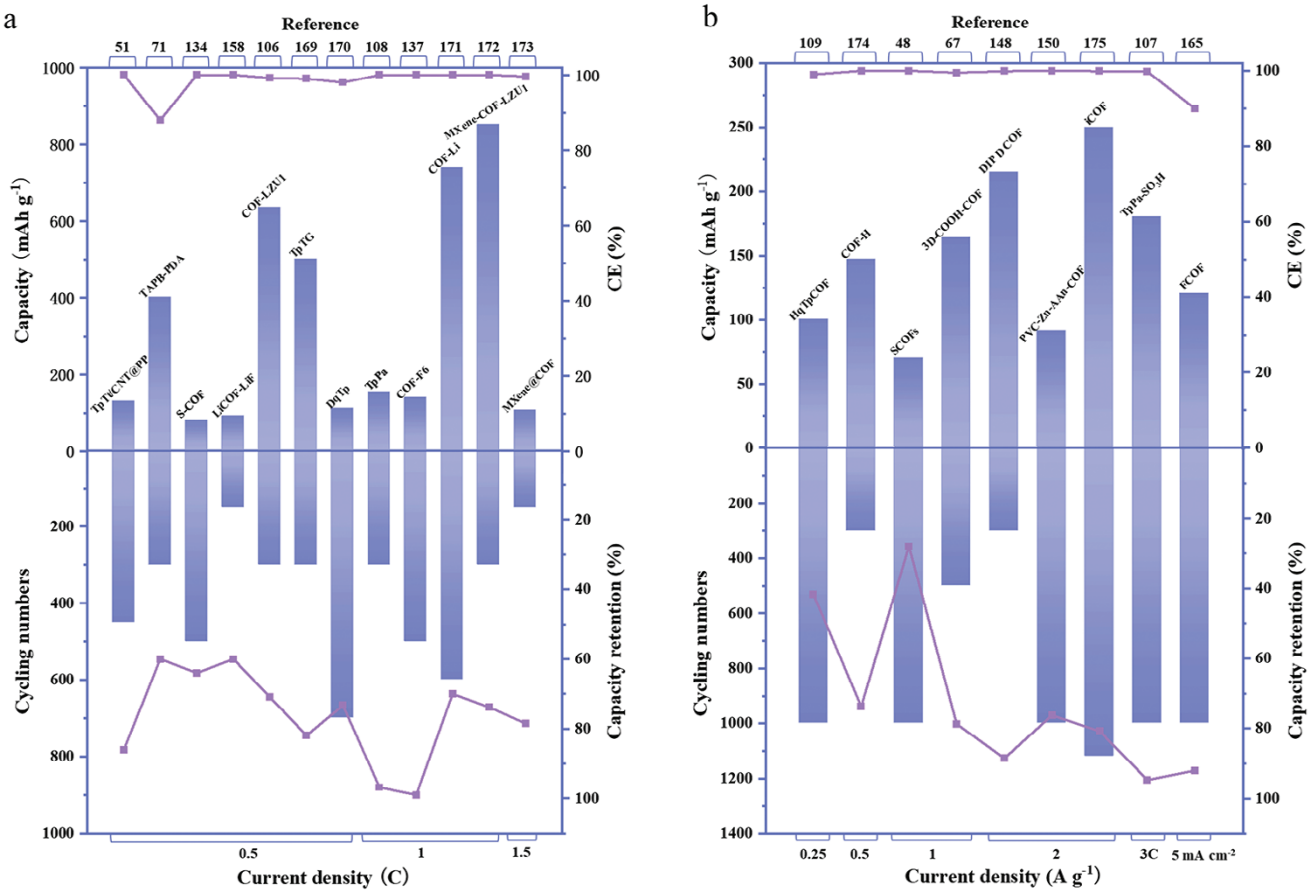
Performance comparison of COF anodes in a) LMBs and b) ZIBs.

**Figure 12:**
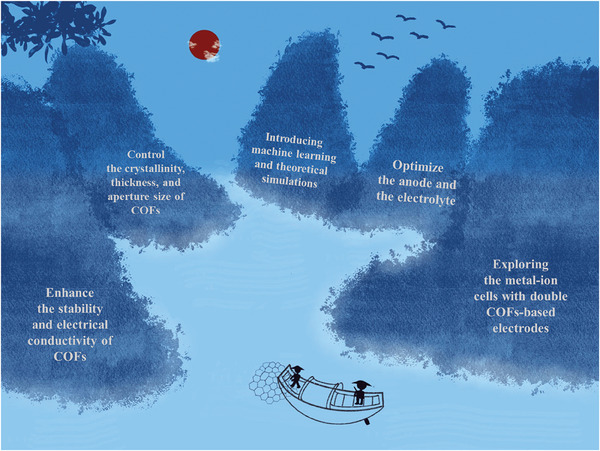
Outlook for the future development of COFs in metal batteries.

Based on previous studies, in this paper, we summarize the types and synthesis methods of COFs and discuss the latest progress and optimization methods of COFs used in Li and Zn anodes to improve battery performance. We aim to understand the challenges faced by metal anodes and the inherent relationship between the rational construction of COF structures and the working mechanism. COFs have the advantages of regular channels, strong designability, and high specific surface area. In the aspect of structural design, electrode materials with better specific capacity can be prepared by enhancing the proportion of reversible redox functional groups in COFs. In addition, the introduction of heteroatoms and continuous conjugated carbon skeleton can effectively enhance the electrical conductivity of the material. In terms of nanostructure design, several approaches can be used to enhance the exposure of the active site. COFs have gained popularity in the research of Li and Zn anodes in rechargeable batteries owing to the above advantages and their attractive electrochemical properties (**Figure** **11**).^[^ ^169^, ^170^, ^171^, ^172^, ^173^, ^174^, ^175^, ^176^ ^]^ The performance of half cells, symmetric cells and full cells with anodes protected by COF has been greatly improved (**Table** **2**). However, numerous challenges still need to be addressed to achieve low‐cost, high‐performance rechargeable metal batteries (**Figure** **12**).

**Table 2:** Summary of the performance of half cells, symmetric cells and full cell with anodes protected by COFs.

Metal anodes	COFs	Half cell		Symmetric cell		Full cell		Ref.
		C/CD. [mA (h) cm^−2^]	CE (%)	C/CD. [mA (h) cm^−2^]	OP. [mV]	Current density	Cycle (times)	
Li (LMBs)	TpTt/CNT@PP	1/‐	>95	1/‐	<15	0.5 C	450	[51]
	TAPB‐PDA	‐	‐	2/1	74	0.5 C	300	[71]
	TpPa	2/2	97.8	5/5	≈50	1 C	300	[108]
	S‐COF	0.5/0.5	97.11	1/1	10.5	0.5 C	500	[134]
	COF‐F6	1/1	98	2/2	≈7.1	1 C	500	[137]
	LiCOF‐LiF	1/1	98.1	10/5	≈80	0.5 C	150	[158]
	COF‐LZU1	0.5/1	99.3	0.5/	≈35	0.5 C	300	[106]
	TpTG	‐	‐	10/1	100	0.5 C	300	[169]
	DqTp	2/1	92	4/1	90	0.5 C	700	[170]
	COF‐Li	5/‐	98.6	10/10	16	1 C	600	[171]
	MXene‐COF‐LZU1	0.5/0.5	97.6	1/1	≈24	1 C	300	[172]
	MXene@COF	1/2	≈99	3/‐	≈125	1.5 C	150	[173]
Zn (ZIBs)	SCOFs	5/1	99.83	5/2	≈40	1 A g^−1^	1000	[48]
	3D‐COOH‐COF	1/1	>99.5	3/3	≈45	1 A g^−1^	500	[67]
	DIP D COF	4/1	99.95	1/1	≈36	2 A g^−1^	300	[148]
	PVC‐Zn‐AAn‐COF	‐	‐	1/20	≈60	2 A g^−1^	1000	[150]
	TAPB‐PDA	‐	‐	5/1	≈62	1 A g^−1^	1000	[133]
	TpPa‐SO_3_H	1/‐	99	1/5	≈25	5 mA cm^−2^	1000	[107]
	FCOF	80/1	97.2	5/1	60	3C	1000	[149]
	HqTpCOF	1/‐	>99	1/1	≈30	250 mA g^−1^	1000	[109]
	COF‐H	2/1	99.2	3/1	22.9	0.5 mA g^−1^	1000	[174]
	iCOF	‐	‐	1/1	≈50	2 A g^−1^	≈1120	[175]
	COF 42	‐	‐	5/5	100	10 mA g^−1^	5000	[176]

Note: Current/ Capacity density (C/CD); Overpotential (OP).

First, anode problems are mutually constrained and often interconnected. Therefore, strategies for addressing these problems should not be isolated. Researchers need to optimize the entire system from a collaborative and comprehensive perspective. In addition, because the electrolyte significantly impacts the anode and even the entire battery, the optimization of the anode–electrolyte interface should be considered when considering the Zn anode protection strategy rather than just modifying the anode surface. However, the current research on electrolytes, including their compositions, in situ state, and compatibility with electrodes, remains in its early stages. Therefore, the coordination relationship between the electrolyte and metal anode is a crucial aspect that must be considered when exploring high‐performance rechargeable metal batteries. Second, theoretical calculations for metal anodes remain insufficient. By establishing the relationship between performance and structure through traditional model building and algorithm, the targeted design and quick screening of organic electrode materials can be achieved. However, it is clear that with the increasing complexity of the problem and the increasing demands on battery performance, the above method can no longer meet the demand. Richer theoretical calculations, accelerating selection of components, reducing the cost of trial and error, using machine learning or theoretical simulation to predict and confirm the active sites of COFs, and understanding the mechanism of free radical transformation during charge and discharge are essential prerequisites for solving the problem. Therefore, it is necessary to introduce more theoretical calculations to provide reliable guidance for optimizing COF‐based electrodes. Third, the synergy of stability and conductivity remains a major obstacle limiting the development of COFs in rechargeable batteries. The current reports of COFs as metal anode protective membranes lack a comprehensive inquiry into the stability. The metal battery with COF film protection has made a great improvement in performance compared with the bare metal battery. However, after more than a thousand cycles, the COF membrane will still be damaged to some extent, thus reducing the protective effect. This may also be one reason why the COF protection metal anode strategy still stuck in the laboratory. Therefore, the high stability of COF plays a crucial role in achieving the commercialization of metal batteries. In addition, compared with conductive carbon materials, such as graphene and CNTs, COFs have lower electrical conductivity due to weak interlayer interactions. The low conductivity of COFs greatly limits the electron transfer rate and reduces the utilization of the active site. Theoretically, reasonable design of highly conjugated structures in layers and strong interactions between layers may be an effective solution for weak interlayer interactions in COFs. For example, hydrogen bonding between layers can increase interlayer interactions. Thus, introducing functional groups (such as ─OH) capable of forming strong hydrogen bonds between different COF layers may be an effective solution, which has rarely been reported. Fourth, the control of the crystallinity, thickness, aperture size, and other parameters of COFs must be considered when COFs are applied to metal anodes. COFs are organic materials with high crystallinity. Appropriate reduction of crystallinity is beneficial to ion conduction. On the metal anode, excessively thick protective film can form a long ion conduction path, which is not conducive to the rapid transport of ions, and excessively thin film may lead to structural dissolution and collapse. Meanwhile, the relatively small pore size can affect the ion flux, while excessively large pore size may facilitate the interaction between the ions and pore wall, thus affecting the normal transport and deposition of ions. Therefore, the adjustment of crystallinity, thickness, and pore size of COFs will greatly mitigate problems faced by metal anodes in LMBs and ZIBs. However, the research on these issues is rarely reported. Finally, COFs can be used as both anode and cathode materials, and notable progress has been made in research regarding their application as cathode materials. The fabrication of metal ion cells containing double COF‐based electrodes is noteworthy.

Despite the challenges, the unique microstructure of COFs endows metal batteries with electrochemical properties, such as ultra‐long cycle life and satisfactory rate performance, which undoubtedly provides a broad road for the commercialization of metal batteries. As a typical organic material, COF electrodes have great potential to decline the cost and environmental pollution of metal batteries. Hence, several challenges and research areas still need to be addressed before the full potential of COFs can be realized in energy storage practical applications. By reviewing and looking forward to these works, we hope to provide some constructive suggestions for the future advancement of COFs in rechargeable batteries.

## Conflict of Interest

The authors declare no conflict of interest.
